# Multi-omics elucidation of recombinant collagen‐mediated modulation of mesenchymal stem cell functions

**DOI:** 10.1016/j.jare.2025.09.032

**Published:** 2025-09-17

**Authors:** Taishan Liu, Juanli Dang, Chenhui Zhu, Xiaoxuan Ma, Linlin Qu, Huan Lei, Daidi Fan

**Affiliations:** a Engineering Research Center of Western Resource Innovation Medicine Green Manufacturing, Ministry of Education, School of Chemical Engineering, Northwest University, Xi’an 710127, China; b Shaanxi Key Laboratory of Biomaterials and Synthetic Biology, Shaanxi R&D Center of Biomaterials and Fermentation Engineering, School of Chemical Engineering, Northwest University, Xi’an 710127, China; c Biotech. & Biomed. Research Institute, Northwest University, Xi’an 710127, China; d Xi’an Giant Biotechnology Co., Ltd., Xi’an 710076, China; e Xi’an Innovative R&D Platform for New Biomedical Materials, School of Chemical Engineering, Northwest University, Xi’an 710127, China; f Department of Plastic and Reconstructive Surgery, Xijing Hospital, Fourth Military Medical University, Xi’an, China

**Keywords:** Recombinant collagen, Mesenchymal stem cell, Multi-omics, Regenerative medicine

## Abstract

**Introduction:**

Regenerative medicine leverages the potential of mesenchymal stem cells (MSCs) and biomaterials to overcome the limitations of traditional organ transplantation. Collagen, a key extracellular matrix component, is widely used, yet its molecular interactions with MSCs remain insufficiently understood.

**Objectives:**

This study investigates how recombinant collagens modulate MSC behavior and paracrine functions to inform the development of bioactive scaffolds for tissue regeneration.

**Methods:**

Recombinant type I and III collagens were biosynthesized using Pichia pastoris. Multi-omics analyses, including transcriptomics and proteomics, were conducted to evaluate the effects of these collagens on MSC gene expression, secretory profiles, and functional impacts on fibroblasts, endothelial cells, and macrophages.

**Results:**

Recombinant collagens regulated key MSC pathways related to angiogenesis, wound healing, adhesion, cytoskeletal organization, and osteogenesis. Proteomic data further revealed enhanced secretion of cytokines and growth factors that influenced the behavior of surrounding stromal and immune cells.

**Conclusion:**

Recombinant collagen, as a bioactive material, can remodel the functions of mesenchymal stem cells. Specifically, recombinant type I collagen primarily activates the *FAK/RHOA/ROCK* signaling pathway, while recombinant type III collagen activates the *PI3K/Akt* signaling pathway; in contrast, bovine-derived type I collagen mainly regulates glycolysis. These findings further support the potential of recombinant collagen in constructing regenerative medicine scaffolds with intelligent regulatory functions.

## Highlights

• Full-length recombinant type I and type III collagen were obtained by fermentation with Pichia pastoris.
• Transcriptomics and proteomics reveal the global effects of recombinant collagen on human mesenchymal stem cells.
• Recombinant collagen affects paracrine effects in mesenchymal stem cells.

## Graphical abstract

**Figure:**
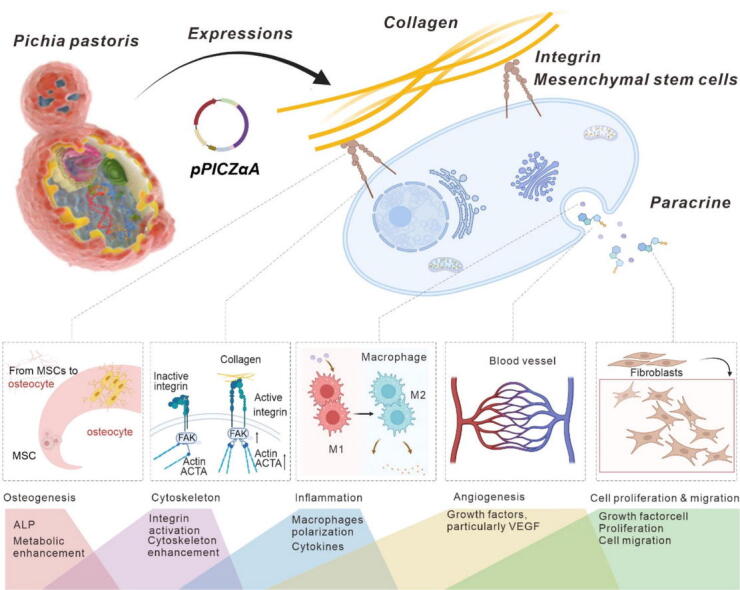

## Introduction

The maintenance, repair, and regeneration of organs and tissues have become critical challenges in regenerative medicine [1]. Mesenchymal stem cells (MSCs) have garnered attention due to their multipotency and paracrine functions [2]. Yet, the short retention time and rapid immune clearance of MSCs after transplantation limit their therapeutic effectiveness [3]. To address this, a variety of natural and synthetic biomaterial scaffolds have been engineered to enhance MSC engraftment, promote cell attachment, and provide structural support for tissue regeneration [4]. Among these, scaffolds derived from natural extracellular matrix (ECM) components—particularly collagen—have demonstrated promising biological compatibility and clinical utility [[5], [6], [7], [8]]. Collagen, a major component of the ECM, plays a crucial role not only in tissue architecture but also in modulating cell behavior [9]. Its interaction with integrins and other cell surface receptors is known to influence adhesion, migration, differentiation, and paracrine signaling [10]. However, despite the widespread application of collagen-based biomaterials, the precise molecular mechanisms through which collagen regulates MSC function—especially in the absence of canonical triple-helical structures—remain poorly understood.

Traditional collagen biomaterials are primarily derived from animal sources, raising safety and ethical concerns, including immunogenicity, pathogen transmission, and batch variability [11,12]. Advancements in genetic engineering and synthetic biology have enabled the production of recombinant human collagen with tunable sequences and improved biosafety, ensuring high purity, reproducibility, and the absence of animal-derived contaminants, addressing key limitations of natural collagen materials used in earlier work [13,14]. Yet, many recombinant collagen products do not retain the full triple-helical structure typical of native collagen [15,16], raising fundamental questions about their ability to recapitulate ECM-mimetic bioactivity. Among collagen types, type I and type III are the most prevalent fibrillar collagens in connective tissues, frequently co-localized in skin, vasculature, and early wound matrices [17]. Type I collagen primarily provides structural integrity and tensile strength, whereas type III collagen plays crucial roles in early repair, tissue elasticity, and microenvironmental remodeling. Notably, the dynamic balance and co-deposition of these two collagens are hallmarks of tissue regeneration processes [18]. Given that mesenchymal stem cells (MSCs) are highly responsive to extracellular matrix (ECM) cues, both type I and III collagens have been implicated in regulating MSC adhesion, migration, and differentiation, as well as angiogenic behaviors in regenerative microenvironments. Some studies have suggested that scaffolds based on collagen (types I and III) can regulate stem cell proliferation and multipotent differentiation [19]. Meanwhile, other studies have shown inconsistent findings, such as reports indicating that type I collagen can reduce keratinocyte differentiation [20] and that type III collagen can enhance cell proliferation and maintain stem cell stemness [21]. Studies also recently indicated that collagen-containing scaffolds can facilitate the differentiation of induced pluripotent stem cells into mesodermal lineages [22]. The findings that type I and III collagen may differentially affect MSC behaviors such as proliferation and differentiation are inconsistent and sometimes contradictory. Furthermore, previous studies have primarily focused on the mechanical and structural properties of collagen scaffolds. Zhang et al. extracted type I collagen from calf skin to modulate MSC chondrogenic differentiation by regulating collagen viscoelasticity [23]; Derek H. Rosenzweig et al. fabricated aligned dense collagen (ADC) hydrogel scaffolds by depositing rat tail type I collagen, inducing tenogenic differentiation of MSCs through mechanical stimulation [24]; Mario L. Fabiilli et al. used rat tail type I collagen to make a collagen scaffold, which guided MSC differentiation toward the osteogenic lineage by ultrasound-induced mechanical strain of the surrounding matrix leading to local radial compression and hardening [25]. These studies have provided relatively limited attention to the biochemical involvement of specific collagen types in cell signaling networks.

**Scheme 1:**
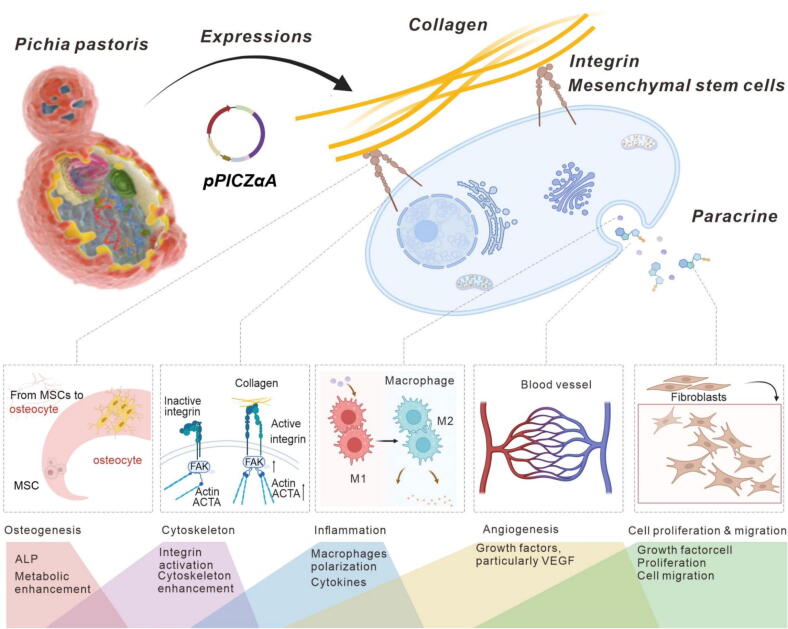
Schematic illustrating how recombinant collagen regulates mesenchymal stem cell behavior and paracrine secretions.

To fill current research gaps, this study adopts an integrated multi-omics strategy to systematically elucidate the molecular mechanisms by which recombinant collagens (type I and III), produced through synthetic biology techniques, regulate the behavior of mesenchymal stem cells (MSCs) in vitro. Scheme 1. Bone marrow mesenchymal stem cells (BMSCs) are the most abundant cells in the bone marrow and are widely present in various connective tissues and organ matrices of the body. They have important application value in tissue engineering and regenerative medicine due to their multidirectional differentiation potential and wide range of biological functions [26]. The investigation focuses on how recombinant collagens influence cell adhesion, cytoskeletal remodeling, and secretory activity; identifies the signaling pathways activated during collagen–MSC interactions, with particular emphasis on integrin-mediated signaling; and further explores whether recombinant collagens lacking a triple-helical structure can still function as bioactive substance. This approach enables us to dissect the distinct biochemical interactions of collagen isoforms with MSCs, providing novel insights for the rational design of collagen-based scaffolds tailored for regenerative medicine applications.

## Results and discussion

### Preparation of recombinant collagen and its effects on stem cells

**Fig. 1:**
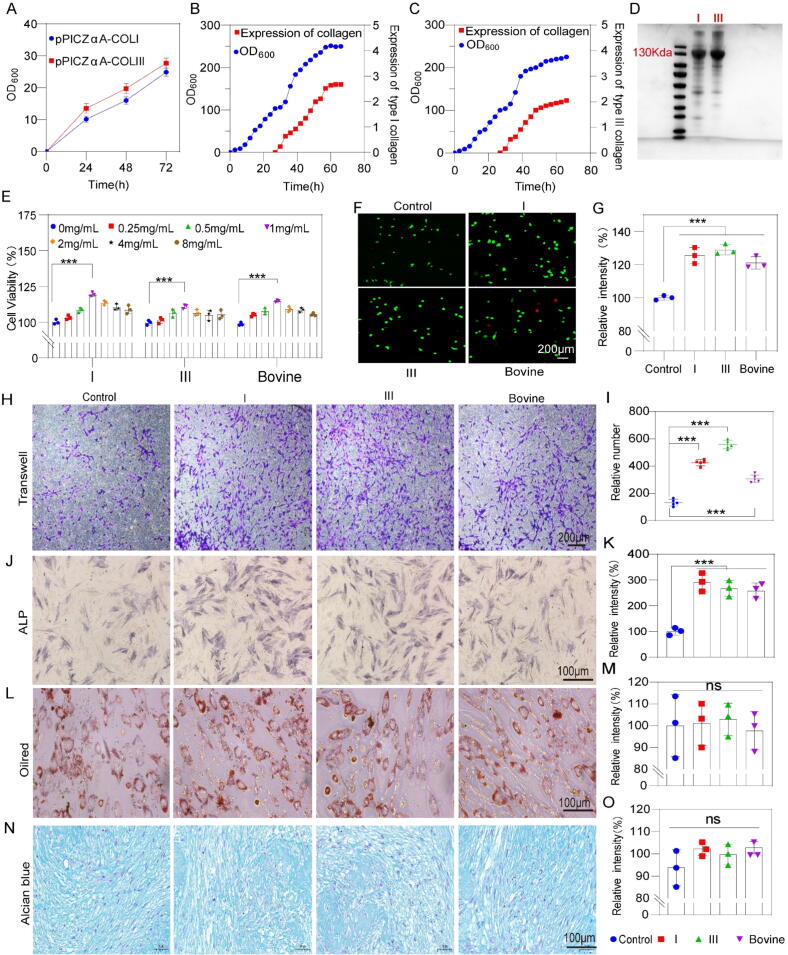
Preparation of recombinant collagen and its effects on stem cells. A) Growth curve of positive transformants over 0–72 h. B–C) Growth and protein expression curves of the strains during scale-up cultivation in a 5 L fermenter. D) SDS-PAGE analysis of the purified recombinant collagen. E) Cell proliferation effects of collagen on mesenchymal stem cells (n = 3). F) Live/dead staining of MSCs after 24 h of different collagen treatments (n = 3), scale bar = 200 µm. G) Alamar Blue assay of different collagen-treated MSCs for 24 h (n = 3). H–I) Transwell assay results and quantification results of MSCs treated with different collagens (n = 3), scale bar = 200 µm. J-K) Representative images and quantitative analysis results of MSCs treated with three collagens stained with alkaline phosphatase (ALP), (n = 3), (scale bar = 100 µm). L-M) Representative images and quantitative analysis results of MSCs treated with three types of collagen stained with Oil Red O (n = 3), (scale bar = 100 µm), N-O) Representative images and quantitative analysis results of MSCs treated with three types of collagen stained with Alizarin Red (n = 3), (scale bar = 100 µm). Significant difference (one-way ANOVA): *P < 0.05, **P < 0.01, and ***P < 0.001.

The gene sequences for the α1 chains of recombinant collagen (types I and III) were introduced into Pichia pastoris (Fig. S1), and high-expressing strains were screened. After 72 h, the OD600 values of the two collagen-expressing strains were 24.86 and 27.73 (Fig. 1A). The large-scale cultivation process in a 5 L fermenter resulted in target protein expression levels of 2.624 g/L (type I) and 2.132 g/L (type III) (Fig. 1B–C). The SDS-PAGE results showed that the molecular weights of the α1 chains of recombinant type I and type III collagens were approximately 110–130 kDa, which was approximately 1.1–1.4 times higher than their theoretical molecular weights (95–100 kDa). This discrepancy could be attributed to the differential migration rates of fibrous collagen molecules in the gel (Fig. 1D). The collagen products underwent digestion using trypsin & Glu-C, elastase, and trypsin & chymotrypsin, after which the products were analyzed using liquid chromatography-tandem mass spectrometry (LC-MS/MS). Comparison with the NCBI database confirmed that recombinant type I collagen produced by fermentation corresponded to the human type I collagen α1 chain fragment 162–1218 (Fig. S2), while recombinant type III collagen corresponded to the human type III collagen α1 chain fragment 154–1221 (Fig. S3). The UV absorption spectra (Figs. S4 and S5) showed that the two recombinant collagens exhibited maximum absorption peaks at 227 and 228 nm, which were consistent with the UV absorption peaks of collagen-like proteins [27]. Using infrared spectroscopy (Fig. S6), C=O bonding telescoping vibrations and N–H bending vibrations were identified at 1629.5–1631.5 cm^−1^, and C-N telescoping and C–H telescoping vibrations were observed at 1542 cm^−1^ and 1239 cm^−1^ in the collagen skeleton, respectively. Both recombinant type I and type III collagens exhibited a distinct negative absorption peak at 198 nm, along with a smaller and broader positive absorption peak at 220 nm (Figs. S7 and S8). These spectral features suggested the presence of irregular coil structures in the secondary structure of the proteins, indicating that recombinant type I and type III collagens did not form the expected triple helix structure.

MSCs were extensively investigated due to their multilineage differentiation potential, paracrine activity, and immunomodulatory properties. Flow cytometric analysis confirmed that the MSCs used in this study were negative for the markers CD34 and CD45, and positive for the mesenchymal markers CD90, CD73, and CD105 (Figs. S9). CCK-8 assay results indicated that the maximal proliferative effect of the three collagen types on MSCs was observed at a concentration of 1 mg/mL (Fig. 1E); thus, this concentration was chosen for subsequent experiments. Live/dead staining of MSCs highlighted the beneficial role of collagen in promoting cell viability (Fig. 1F). The Alamar Blue assay results indicated that recombinant type I, recombinant type III, and bovine type I collagens enhanced the metabolic activity of MSCs (Fig. 1G). Additionally, the Transwell migration assay results confirmed that these three types of collagen significantly improved the migration capability of bone marrow-derived MSCs (Fig. 1H–I). The expression of alkaline phosphatase (ALP) was enhanced in all three collagens (Fig. 1J-K), underscoring the crucial role of collagen in guiding osteogenic differentiation. However, no significant differences were detected in lipid droplet formation or glycosaminoglycan production among the four groups (Fig. 1L–O), indicating that the three collagens were ineffective in guiding the adipogenic and chondrogenic differentiation of MSCs.

### Collagen-induced actin cytoskeleton organization

**Fig. 2:**
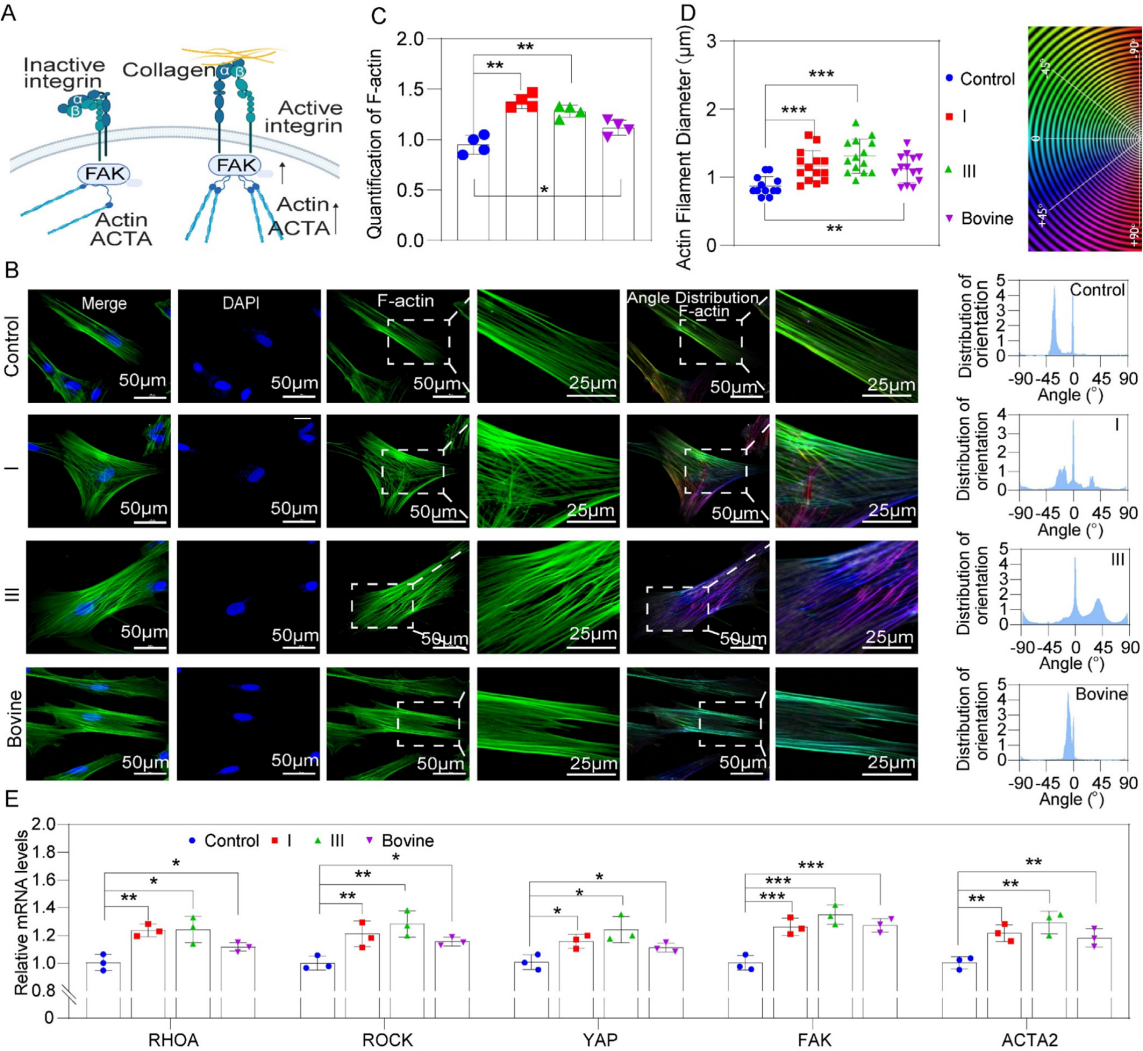
Collagen-induced actin cytoskeleton organization. A) Schematic depicting the possible interactions between collagen and the integrin molecules on the cell surface. B) Confocal immunofluorescence images of *F-actin* polymerization. C). Quantitative analysis of *F-actin* localization in mesenchymal stem cells (n = 4). D) Quantification of the average actin fiber diameter (n = 15). E) qPCR data results for actin-related gene expression (n = 3). Significant difference (one-way ANOVA): *P < 0.05, **P < 0.01, and ***P < 0.001.

The transcription levels of integrins in MSCs were assessed following collagen treatment (Figs. S10–S11). The results indicated that recombinant type I collagen significantly enhanced the gene expression of several integrins, including *ITGA1, ITGA6, ITGA8, ITGA9, ITGB3, ITGB7,* and *ITGA9*. Similarly, recombinant type III collagen significantly upregulated the expression of integrins such as *ITGA1, ITGA2, ITGA3, ITGB4, ITGA6, ITGA1, ITGA3*, and *ITGA8*. These results indicated that the effects of recombinant collagens on the cells were possibly influenced by integrin signaling (Fig. 2A). Integrins are a family of glycoproteins composed of non-covalently linked α- and β-subunits, which serve as the main transmembrane adhesion receptors that connect the ECM to the cytoskeleton, and are essential for ECM adhesion and ECM-receptor interactions [28,29]. The organization of the cytoskeleton plays a pivotal role in initiating signaling cascades at the cell–material interface [30]. Mesenchymal stem cells (MSCs) actively reorganize their filamentous actin (F-actin) network and generate cytoskeletal tension in response to extracellular cues [31]. F-actin is a dynamic filamentous system that undergoes continuous polymerization–depolymerization cycles, regulating cell adhesion, morphology, and migration in accordance with the local microenvironment [32,33]. Its abundance and organization directly influence cell stiffness and osteogenic differentiation potential, and inhibition of F-actin depolymerization has been shown to enhance bone formation [34,35]. Following treatment with different collagen types, MSCs exhibited a markedly broader actin leading edge, with actin assembly predominantly localized at the cell periphery, suggesting that filament network polymerization was triggered toward the plasma membrane. Quantitative analysis revealed a 37 %, 27 %, and 10 % increase in cortical actin ratio for recombinant type I collagen, recombinant type III collagen, and bovine type I collagen, respectively (Fig. 2B–C). Notably, the extent of actin filament elongation across the cytoplasm strongly correlated with osteogenic differentiation [36].

It is well established that actin filament orientation plays a critical role in stem cell fate decisions. For example, during osteogenic differentiation of MSCs, cytoskeletal remodeling is consistently accompanied by parallel alignment and orientation of actin filaments [37]. Orientation analysis using Orientation J (Biomedical Imaging Group, Switzerland) confirmed that collagen treatment induced substantial cytoskeletal reorganization and filament alignment. Moreover, the average actin filament diameters in the control, recombinant type I collagen, recombinant type III collagen, and bovine type I collagen groups were 0.87 μm, 1.16 μm, 1.31 μm, and 1.08 μm, respectively (Fig. 2D). The increase in filament diameter indicates that collagen exposure enhanced cytoskeletal tension in MSCs, a condition previously reported to favor osteogenic differentiation [38,39].

To elucidate the underlying mechanism, we performed quantitative PCR analysis of cytoskeleton remodeling–related genes (*FAK*, *ACTA2*, *ROCK*, *RHOA*, and *YAP*) (Fig. 2E). The results demonstrated that collagen-induced cytoskeletal alignment was associated with FAK activation and stimulation of the *RHOA/ROCK* signaling pathway.

### Comprehensive transcriptomic changes induced by collagen in stem cells

**Fig. 3:**
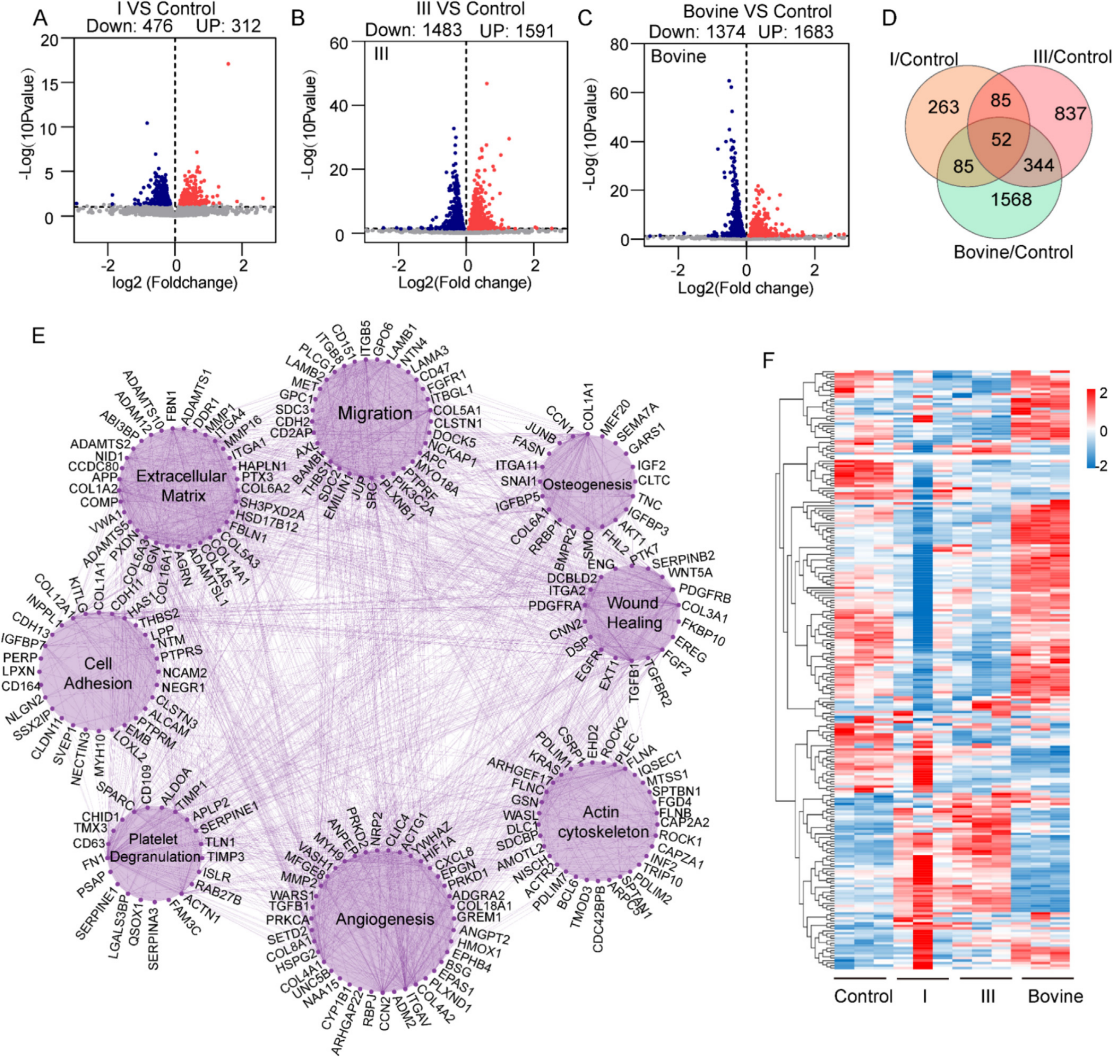
Comprehensive transcriptomic analysis of MSCs treated with collagen proteins. A–C) Volcano plot of gene expression changes in MSCs with different collagen treatments. D) Venn diagram of differentially expressed genes (DEGs). E) Co-expression interaction network of DEGs generated from Cytoscape analysis, highlighting the effects of collagen proteins on MSC populations. F) Heatmap of DEGs encoding secreted proteins. Significant difference (one-way ANOVA): *P < 0.05, **P < 0.01, and ***P < 0.001.

To explore the extensive transcriptomic changes in MSCs treated with various types of collagen for 3 days, RNA-seq analysis was conducted (Fig. S12). Gene expression levels were normalized to fragments per kilobase of transcript per million mapped reads (FPKM) for each gene across all samples. The gene expression levels (FPKM) between the replicates under different conditions showed high consistency, as follows: control (0.998 < r < 1), recombinant type I collagen (0.985 < r < 1), recombinant type III collagen (0.999 < r < 1), and bovine type I collagen (0.998 < r < 1) (Fig. S13). The recombinant type I collagen group exhibited 312 upregulated and 476 downregulated differentially expressed genes (DEGs) (Fig. 3A).

In the recombinant type III collagen group, 1591 genes were markedly upregulated, while 1483 genes were notably downregulated (Fig. 3B). Conversely, in the bovine type I collagen group, 1374 genes exhibited significant upregulation, and 1683 genes demonstrated significant downregulation (Fig. 3C). These results indicated that treatment with recombinant type I, recombinant type III, and bovine type I collagens produced a strong cellular response in MSCs. The overlap of the DEGs between pairwise comparisons revealed only 52 common DEGs, with 263 DEGs changing in the recombinant type I collagen group, 837 in the type III collagen group, and 1568 in the bovine type I collagen group, highlighting the distinct but significant effects of these collagen types on MSCs (Fig. 3D). These findings strongly suggested that treatment with recombinant type I, recombinant type III, and bovine type I collagens induced significant cellular responses in MSCs, potentially through different underlying mechanisms as indicated by the DEGs.

To identify the pathways significantly affected by collagen treatment, Cytoscape [40] and the String database (https://cn.string-db.org) were used to group genes based on known pathways and signaling functions. This approach created a gene network to visualize the genetic interdependencies among the DEGs (p < 0.05) (Fig. 3E). The analysis revealed eight distinct clusters related to cellular processes and functions from the DEG dataset, namely, “angiogenesis,” “wound healing,” “cell adhesion,” “cell migration,” “platelet degranulation,” “cytoskeleton,” “ECM organization,” and “osteogenic differentiation.”.

To further investigate the paracrine secretions [41,42], the expression levels of 3947 genes encoding secretory proteins were analyzed using RNA-seq, and the list of 3947 secretory protein-encoding genes was sourced from The Human Protein Atlas (https://www.proteinatlas.org/). Among these, 191 genes showed differential expression across the four treatment groups (Fig. 3F). Notable variations were detected, including inflammatory factors, ECM components, growth factors, and matrix metalloproteinases (Fig. S14). These factors played critical roles in regulating biological processes related to tissue repair and regeneration, including matrix metalloproteinases, angiogenesis, immune modulation, and ECM remodeling. Treatment with the three collagens not only induced the osteogenic differentiation of MSCs but also modulated paracrine secretions, offering valuable insights into the therapeutic benefits of collagen in regenerative medicine.

### Effects of recombinant type I collagen on bone marrow mesenchymal stem cell function

**Fig. 4:**
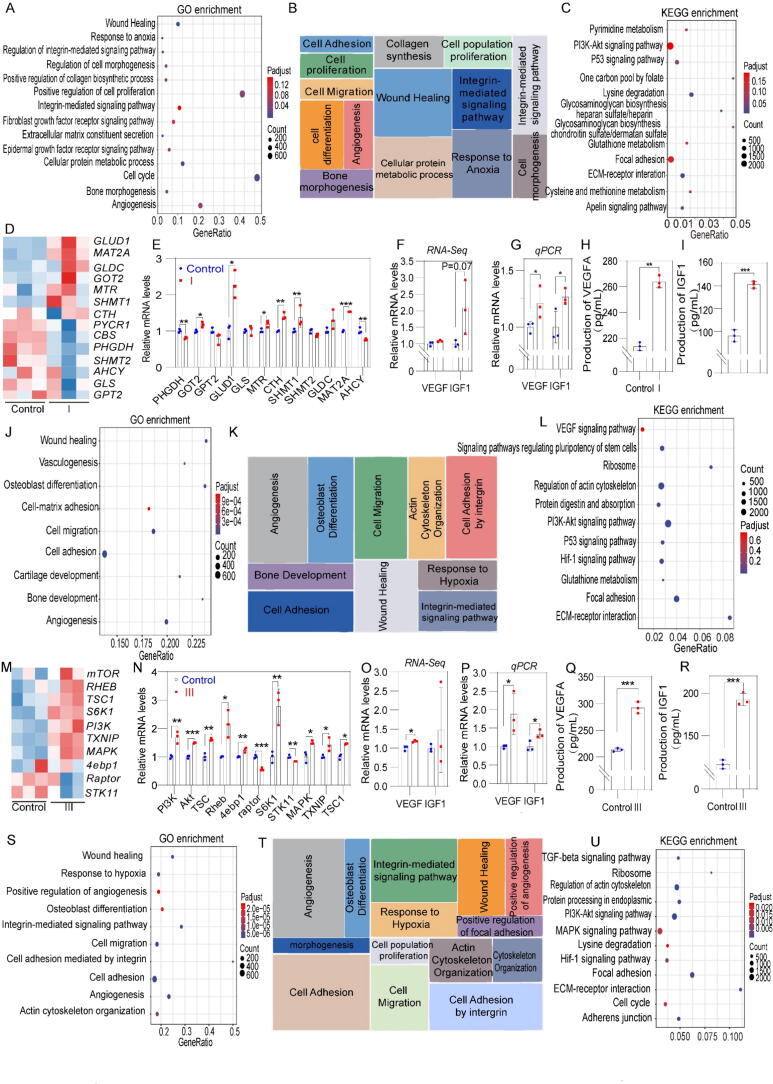
Functional analysis of collagen-induced changes in bone marrow mesenchymal stem cells. A) Gene Ontology (GO) terms of biological processes significantly enriched in DEGs upregulated by type I collagen treatment. B) REduce + VIsualize Gene Ontology (REVIGO) analysis of enriched GO terms enriched by type I collagen treatment. C) Enrichment analysis of KEGG pathways upregulated by type I collagen treatment, D) Heatmap of gene expression levels related to amino acid metabolism after type I collagen treatment. E) mRNA levels of amino acid metabolism-related genes after type I collagen treatment (n = 3). F-G) Expression of vascular endothelial growth factor (*VEGFA*) and insulin-like growth factor 1 (*IGF1*) in MSCs after type I collagen treatment by RNA-seq and qPCR. H-I) Enzyme-linked immunosorbent assay (ELISA) measurements of *VEGFA* and *IGF1* levels (n = 3). J) GO terms of biological processes significantly enriched in DEGs upregulated by type III collagen treatment. K) REVIGO analysis of enriched GO terms enriched by type III collagen treatment. L) Enrichment analysis of KEGG pathways upregulated by type III collagen treatment. M) Heatmap of gene expression levels related to the *PI3K/AKT* pathway after type III collagen treatment. N) *PI3K/AKT* pathway-related gene expression levels after type III collagen treatment (n = 3). O-P) Expression of *VEGFA* and *IGF1* in MSCs after type III collagen treatment. Q-R) ELISA measurements of *VEGFA* and *IGF1* levels (n = 3). S) GO terms of biological processes significantly enriched in DEGs upregulated by Bovine type I collagen treatment. T) REVIGO analysis of enriched GO terms after bovine type I collagen treatment. U) Enrichment analysis of KEGG pathways upregulated by bovine type I collagen treatment. Significant difference (one-way ANOVA): *P < 0.05, **P < 0.01, and ***P < 0.001.

To assess the effect of recombinant type I collagen on MSCs, Gene Ontology (GO) enrichment analysis of the upregulated DEGs was conducted using a BGI multi-omics system (https://biosys.bgi.com/). This analysis identified several prominent GO terms in the biological processes (BP), as illustrated in Fig. 4A, highlighting the significant effects of recombinant type I collagen on MSC functions, which consisted of “wound healing,” “integrin-mediated signaling pathway,” “collagen biosynthesis,” “cellular response to hypoxia,” “osteoblast differentiation,” “angiogenesis,” and “positive regulation of cell proliferation”. These findings suggested that recombinant type I collagen modulated multiple MSC functions, including “collagen synthesis and secretion,” “angiogenesis,” “osteogenic differentiation,” and “adaptation to hypoxia”.

REVIGO analysis was performed to group the enriched biological process GO terms into several categories, including “collagen biosynthetic process,” “wound healing,” “osteoblast differentiation,” “cellular response to hypoxia,” and “cell adhesion” (Fig. 4B). These clusters, which demonstrated significant enrichment and were highly pertinent to the identified biological processes, validated the observed changes in bone marrow MSCs. The findings indicated that integrin-mediated signaling pathways, angiogenesis, and wound healing were the key cellular responses of MSCs to recombinant type I collagen treatment.

Kyoto Encyclopedia of Genes and Genomes (KEGG) enrichment revealed several potential signaling pathways activated by recombinant type I collagen in MSCs, namely, “*ECM*-receptor interaction,” “chondroitin sulfate biosynthesis,” “heparan sulfate biosynthesis,” “*p53* signaling,” “glycine, serine, and threonine metabolism,” “sphingolipid metabolism,” “cysteine and methionine metabolism,” “glutathione metabolism,” “pyrimidine metabolism,” “cell-matrix adhesion,” and “*HIF-1α* signaling” (Fig. 4C).

The expression of amino acid metabolism-related genes in MSCs treated with recombinant type I collagen was subsequently evaluated. Transcriptomic profiling using RNA-seq (Fig. 4D–E), followed by qPCR validation, demonstrated that genes associated with amino acid metabolism, such as *GLUD1, MAT2A, GLDC, MTR, SHMT1,* and *CTH*, were upregulated in the recombinant type I collagen group, which suggested that the significant effect of recombinant type I collagen on MSCs may be mediated by changes in amino acid metabolism-related pathways.

Collagen is a core component of the extracellular matrix (ECM), and its biosynthesis depends on the supply of specific amino acids [43]. The catabolism of glutamine and serine is key to the synthesis of proline and glycine, respectively, two essential building blocks of collagen. To investigate the role of glutamine and serine metabolism in ECM remodeling, we inhibited glutaminase (*GLS1*, an enzyme that catalyzes the conversion of glutamine to glutamate, the first step in proline synthesis) and serine hydroxy methyltransferase (*SHMT1*, an enzyme that catalyzes the conversion of serine to glycine) [44]. Inhibition of *GLS1* activity with the small molecule inhibitor CB-839 significantly reduced proline biosynthesis, while inhibition of *SHMT1* activity with SHIN1 blocked glycine production. The results showed that inhibition of either *GLS1* or *SHMT1* resulted in decreased collagen expression in the ECM, suggesting that glutamine and serine catabolism play a crucial role in regulating collagen synthesis and ECM remodeling (Fig. S15).

Treatment with recombinant type I collagen noticeably improved the ability of MSCs to promote angiogenesis. RNA-seq analysis revealed an upregulation of vascular endothelial growth factor (*VEGFA*) (8 %) and insulin-like growth factor 1 (*IGF1*) (108 %, p = 0.07) in the recombinant type I collagen group compared with the control. These findings were further validated by qPCR, which showed significant increases in *VEGFA* (21 %, p < 0.05) and *IGF1* (26 %, p < 0.05) expression (Fig. 4F-G). Enzyme-linked immunosorbent assay (ELISA) confirmed that MSCs treated with recombinant type I collagen secreted significantly higher levels of *VEGFA* (263.9 pg/mL, p < 0.01) and *IGF1* (161.04 pg/mL, p < 0.001) compared with control group (Fig. 4H-I). *VEGFA* serves as a critical angiogenic factor, with elevated levels capable of effectively inducing angiogenesis [[45], [46], [47]]. Additionally, *IGF1* was found to promote angiogenesis through the activation of the *PI3K/Akt* signaling pathway [48]. Therefore, MSCs treated with recombinant type I collagen possibly enhanced angiogenesis in the surrounding stem/progenitor and endothelial cells by secreting *VEGFA* and *IGF1*.

### Effects of recombinant type III collagen on bone marrow mesenchymal stem cell function

GO analysis revealed that recombinant type III collagen activated several notable biological processes in MSCs, including “angiogenesis,” “cell migration,” “osteogenic differentiation,” “cell adhesion,” “integrin-mediated signaling pathway,” “vascular deposition,” and “response to hypoxia” (Fig. 4J). These findings suggested that recombinant type III collagen has the potential to regulate various MSC functions.

In addition, REVIGO analysis refined the enriched GO terms into key clusters, including “angiogenesis,” “osteogenic differentiation,” “cell migration,” “cell adhesion,” “cytoskeleton regulation,” and “response to hypoxia.” These results demonstrated that recombinant type III collagen significantly enhanced the osteogenic differentiation, cell adhesion, and angiogenic capabilities of MSCs (Fig. 4K).

KEGG enrichment analysis indicated that recombinant type III collagen potentially activated several MSC signaling pathways, including “*ECM*-receptor interaction,” “focal adhesion,” “*PI3K/Akt*,” “cytoskeleton regulation,” and “*AMPK* signaling.” The integrin family, functioning as the main transmembrane adhesion receptors connecting the ECM to the cytoskeleton, has been shown to be crucial for ECM adhesion and interactions between the ECM and receptors [49]. Notably, the *PI3K/Akt* pathway serves as a downstream pathway of integrin signaling [50]. Therefore, the expression of *PI3K/Akt*-related genes was further evaluated in MSCs treated with recombinant type III collagen. In the transcriptome profile of MSCs obtained through RNA-seq, which was further validated by qPCR (Fig. 4L-M), the recombinant type III collagen group showed an increased expression of *PI3K/Akt*-related genes, including *PI3K* (73 %), *AKT* (50 %), *TSC* (65 %), *Rheb* (114 %), and *MAPK* (46 %) (Fig. 4N). This result suggested that recombinant type III collagen exerted a significant influence on MSCs, likely by activating the *PI3K/Akt* pathway. Consistently, immunofluorescence staining consistently showed that the phosphorylation of both FAK and AKT was increased in MSCs co-cultured with recombinant type III collagen (Fig. S16), providing direct evidence for integrin activation and the involvement of the downstream *PI3K/Akt* pathway.

GO analysis revealed that enhanced angiogenesis was a key cellular response to recombinant type III collagen treatment in MSCs. RNA-seq and qPCR data demonstrated a significant upregulation of angiogenesis-related genes, such as *VEGFA* and *IGF1* (p < 0.05) (Fig. 4O-P). Additionally, the ELISA results showed a substantial increase in the secretion levels of *VEGFA* (292.3 pg/mL, p < 0.001) and *NGF* (192.5 pg/mL, p < 0.001) in the recombinant type III collagen group (Fig. 4Q-R).

### Effects of bovine type I collagen on bone marrow mesenchymal stem cell function

Bovine type I collagen activated several significant biological processes in MSCs, including “cell adhesion,” “extracellular matrix remodeling,” “angiogenesis,” “integrin-mediated signaling pathways,” “cell migration,” “wound healing,” “cell response to hypoxia,” “cytoskeletal changes,” “osteogenic differentiation,” “vascular remodeling,” “cell-matrix adhesion,” and “cell proliferation” (Fig. 4S). These results suggested that bovine type I collagen could modulate various MSC functions, including angiogenesis, osteogenic differentiation, and cell proliferation and migration.

REVIGO analysis categorized biological process GO terms into groups, such as “angiogenesis,” “osteogenic differentiation,” “cell adhesion,” “response to hypoxia,” and “wound healing” (Fig. 4T). These clustered terms exhibited a strong correlation with the original biological process GO terms and demonstrated high significance, validating the identified changes in bone marrow MSCs. The results notably highlighted that angiogenesis, cell adhesion, and wound healing were the key cellular responses of MSCs to bovine type I collagen treatment.

KEGG enrichment analysis identified several signaling pathways potentially activated by bovine type I collagen treatment in MSCs, including “*ECM*-receptor interaction,” “focal adhesion,” “cytoskeletal regulation,” and “*HIF-1α* signaling pathway.” Notably, *HIF-1α* signaling was found to be closely associated with these processes (Fig. 4U). Additional evaluation of *HIF-1α*-related gene expression in MSCs treated with bovine type I collagen was conducted. RNA-seq data (Fig. S17A-B) and subsequent qPCR validation showed the upregulation of several glycolysis-related genes, including *PDK1* (23.1 %), *ENO1* (18.5 %), *HK1* (34.8 %), *PFKFB3* (58.1 %), *LDHA* (21.2 %), and *SLC2A1* (65.2 %), compared with the control group. Excessive *HIF-1α* has been shown to shift oxidative phosphorylation to glycolysis, leading to compromised cellular function [51]. The qPCR results further confirmed the upregulation of *HIF-1α* RNA (1.39-fold), suggesting that the significant effect of bovine type I collagen on MSCs possibly involved a shift to glycolysis through the *HIF-1α* signaling pathway. GO analysis indicated that enhanced angiogenesis was a key cellular event following the treatment of MSCs with bovine type I collagen. RNA-seq and qPCR data demonstrated significant upregulation of angiogenesis-related genes, including *VEGFA* and *IGF1*, in the bovine type I collagen-treated group (p < 0.05) (Fig. S17C-D). Additionally, ELISA showed significantly higher secretion levels of *VEGFA* (284.5 pg/mL, p < 0.001) and *IGF1* (220.3 pg/mL, p < 0.001) in the bovine type I collagen-treated group compared with control group (Fig. S17E-F).

Three collagens significantly influenced various biological processes and pathways in MSCs. Specifically, recombinant type I collagen enhanced ECM remodeling and angiogenesis in MSCs by modulating the amino acid metabolism pathways and increasing the secretion of angiogenic factors. Meanwhile, recombinant type III collagen effectively induced osteogenic differentiation in MSCs by activating the *PI3K/AKT* pathway, which in turn enhanced the secretion of angiogenic factors and stimulated angiogenesis. Bovine type I collagen enhances glycolysis through HIF-1α regulation, thereby promoting angiogenesis in mesenchymal stem cells.

### Changes in the secretome of collagen-induced mesenchymal stem cells

**Fig. 5:**
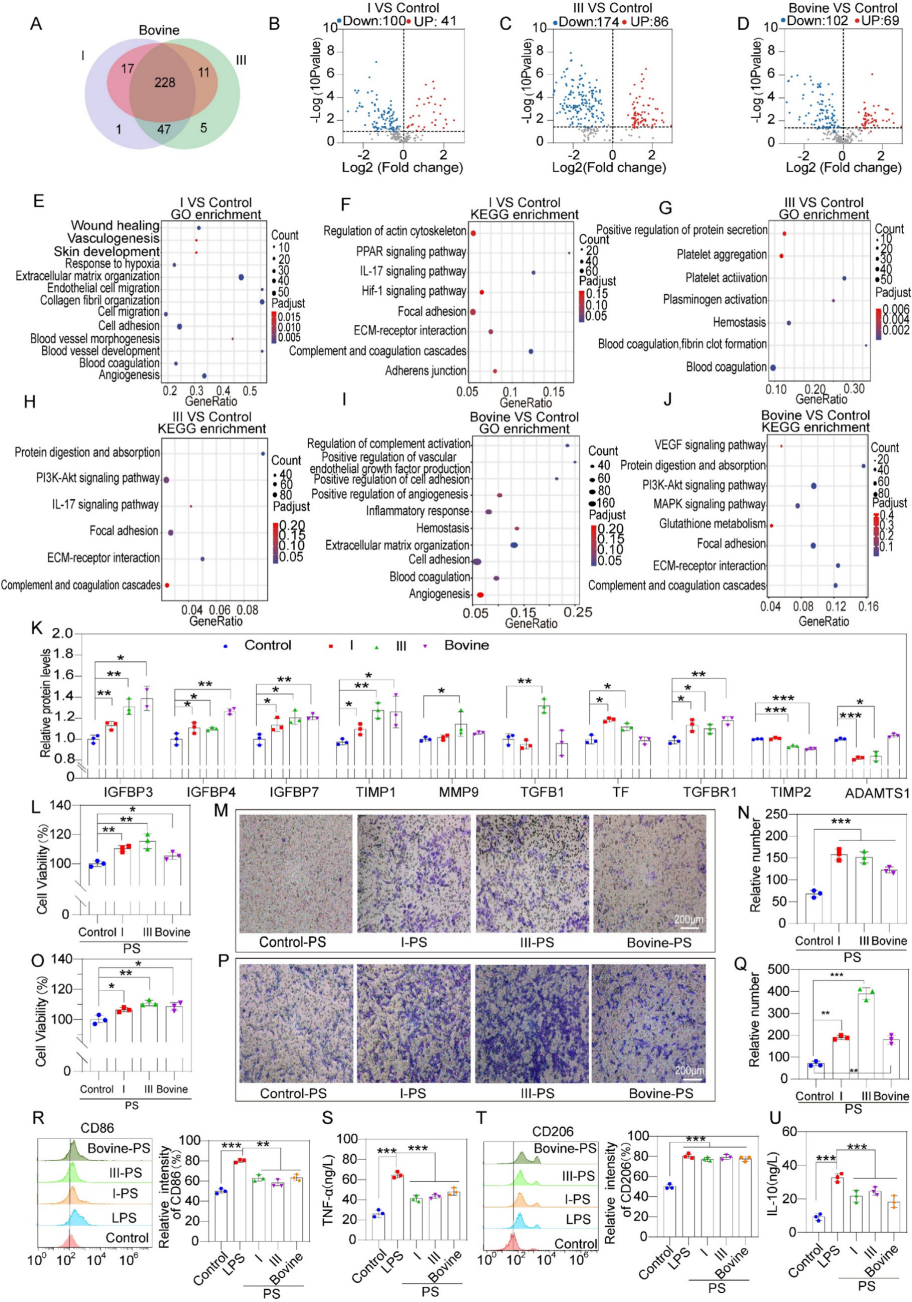
Changes in the mesenchymal stem cell secretome and regulation of cellular behavior induced by collagens. A) Venn diagram of secretory proteins in supernatants. B–D) Volcano gram of secreted proteins in the supernatant. E–F) GO and KEGG enrichment results for significantly altered DEPs in MSCs treated with recombinant type I collagen. G–H) GO and KEGG enrichment results for significantly altered DEPs in MSCs treated with recombinant type III collagen. I–J) GO and KEGG enrichment results for significantly altered DEPs in MSCs treated with bovine type I collagen. K) Proteomic analysis revealed secreted factors related to collagen, growth factors, and matrix metalloproteinases (n = 3). L) CCK-8 data of human umbilical vein endothelial cells (HUVECs) treated with different groups of PS for 24 h (n = 3). M−N) Transwell assay of HUVECs treated with different groups of PS (n = 3), scale bar = 200 µm. O) CCK-8 data of human skin fibroblast (HSF) treated with different groups of PS for 24 h (n = 3). P-Q) Transwell assay of HSF treated with different groups of PS for 24 h (n = 3), scale bar = 200 µm. R) Flow cytometry analysis of CD86-positive (M1 type) THP-1 cells treated with LPS, along with quantification. S) TNF-α content of the THP-1 supernatant incubated for 24 h with different groups of PS (n = 3). T) Flow cytometry analysis of CD206-positive (M2 type) THP-1 macrophages treated with IL-4, along with quantification. U) IL-10 content of the THP-1 supernatant incubated for 24 h with different groups of PS (n = 3). Significant difference (one-way ANOVA): *P < 0.05, **P < 0.01, and ***P < 0.001.

Proteomic analyses of the cell supernatants after different collagen treatments were performed to investigate changes in the secretome, as shown in Fig. S18. A Venn diagram of the secretome proteomics data is shown in Fig. 5A. Analysis revealed that the MSCs across all four treatment groups shared a highly similar secretome, identifying 228 common proteins. This suggested that the three collagens predominantly induced changes in the levels of secreted proteins, rather than altering the types of secreted proteins. Specifically, 141 differentially expressed proteins (DEPs) were identified between recombinant type I collagen and the control (p < 0.05), with 41 proteins upregulated and 100 downregulated (Fig. 5B). In the case of recombinant type III collagen versus the control, 260 DEPs were identified (p < 0.05), with 86 proteins upregulated and 174 downregulated (Fig. 5C). By contrast, the bovine type I collagen group exhibited 69 significantly upregulated proteins and 102 significantly downregulated proteins (Fig. 5D).

Functional insights into these secretome changes were obtained through GO and KEGG enrichment analyses (Fig. 5E–J). A comparison between recombinant type I collagen and the control indicated specific biological processes, such as “angiogenesis,” “cellular response to hypoxia,” “cell migration,” “wound healing,” and “cell adhesion” (Fig. 5E). These results strongly indicated the significant potential of paracrine signals induced by recombinant type I collagen in angiogenesis and tissue regeneration. KEGG analysis revealed that the altered secretome in the recombinant type I collagen group was linked to several cellular pathways, including “*ECM*-receptor interaction,” “focal adhesion,” “*PI3K/Akt* signaling pathway,” “*IL-17* signaling pathway,” and “complement and coagulation cascades” (Fig. 5F).

A comparison between recombinant type III collagen and control groups identified specific biological processes, including “*ECM* remodeling,” “regulation of hemostasis activation,” “positive regulation of *VEGF* production,” “cell adhesion,” “angiogenesis,” “hemostasis,” and “cell adhesion” (Fig. 5G).

These findings strongly suggested that the significant potential in ECM remodeling and hemostasis was provided by paracrine secretions induced by recombinant type III collagen. KEGG analysis indicated that the altered secretome in the recombinant type III collagen group was associated with various cellular pathways, including “complement and coagulation cascades,” “*IL-17* signaling pathway,” “*PPAR* signaling pathway,” “*ECM*-receptor interaction,” and “focal adhesion” (Fig. 5H).

In the bovine type I collagen versus control comparison, the identified biological processes included “platelet activation,” “coagulation,” “platelet activation,” “platelet aggregation,” and “wound healing” (Fig. 5I). These results indicated that substantial potential in hemostasis was provided by paracrine secretions induced by bovine type I collagen. KEGG analysis further revealed that the altered secretome in the bovine type I collagen group was linked to several cellular pathways, including “*PI3K/Akt* signaling pathway,” “protein digestion and absorption,” “focal adhesion,” “*ECM*-receptor interaction,” “*MAPK* signaling pathway,” “*VEGF* signaling pathway,” and “glutamine metabolism” (Fig. 5J).

After treatment with different collagens, the MSCs exhibited signaling pathways in their secretomes, which were enriched and closely matched the RNA sequence data (Fig. 4C, J, and Q). The RNA-seq results indicated significant changes in genes related to matrix metalloproteinases and growth factors, prompting an investigation into the secretion levels of relevant proteins (Fig. 5K). The observed changes in protein expression and secretion aligned with the RNA-seq data. Three collagen groups demonstrated significantly higher levels of growth factors compared with control group, including insulin-like growth factor binding proteins *IGFBP3, IGFBP4*, and *IGFBP7*, as well as *TGFBR1* and *TIMP1*. Additionally, *TIMP2* was reduced by 9 % and 7 % of the original levels in the recombinant type III collagen and bovine type I collagen treatments, respectively. *ADAMTS1* decreased by 19 % and 13 % in the recombinant type I and type III collagen treatments, respectively. The recombinant type III collagen group showed increases in *TGFB1* and *MMP9* by 31 % and 14 %, respectively.

### Mesenchymal stem cell stimulated by recombinant collagen produce paracrine signals that regulate fibroblasts, HUVECs, and macrophages

The CCK-8 assay showed a significant increase in human umbilical vein endothelial cell (HUVEC) proliferation when HUVECs were cultured with PS derived from all three collagen-treated MSCs (Fig. 5L). After 24 h of culture, all three types of collagen induced PS, which enhanced HUVEC migration, and bovine type I collagen demonstrated the smallest effect (Fig. 5M and N). This suggested that paracrine secretions from MSCs in response to collagen enhanced the migration and proliferation of HUVECs, highlighting the substantial potential of these signals in promoting angiogenesis.

Human skin fibroblasts (HSFs) treated with collagen-induced PS exhibited increased proliferation rates at 24 h, suggesting that these paracrine secretions from collagen-treated MSCs play an active role in promoting fibroblast proliferation (Fig. 5O). The Transwell assay results demonstrated that PS induced by all three types of collagen promoted HSF cell migration, with PS from recombinant type III collagen showing the most pronounced effect (Fig. 5P-Q). In summary, these findings suggested that paracrine secretions from collagen-treated MSCs could promote fibroblast proliferation and migration.

Macrophage phenotypes were assessed using classical markers for M1 (*CD86*) and M2 (*CD206*) macrophages via flow cytometry [52]. As shown in Fig. 5R and T, the flow cytometry results revealed significantly fewer *CD86*-positive cells in the three collagen PS groups than in the LPS group (100 ng/mL). Conversely, these PS groups exhibited *CD206*-positive cells at levels comparable to those in the 100 ng/mL *IL-4* treatment group. LPS was used to induce M1 macrophage polarization, and the interaction between MSC and collagen was used in a co-culture system to study the effect of MSC and collagen on M1-M2 macrophage polarization. CD86/CD206 immunofluorescence staining results showed that the secretomes produced by the three collagen-induced stem cell paracrine pathways can promote macrophage M1-M2 polarization (Fig. S19). Additionally, the ELISA results indicated that PS induced by recombinant collagens markedly decreased TNF-α secretion while increasing IL-10 secretion in macrophages (Fig. 5S and U). Collectively, these findings indicated that paracrine secretions from MSCs, stimulated by recombinant collagen, could promote macrophage polarization toward the M2 phenotype.

To further elucidate the mechanisms behind macrophage polarization induced by MSC-derived paracrine secretions, MSCs treated with collagen were examined to reveal the different expression levels of several inflammatory genes, including *TNFSF15, IL6, IL17D, IL32, CSF2RA, IL2RB, TNFSF9*, and *CXCL12*, among the different groups (Fig. S13) [53]. Notably, the expression levels of anti-inflammatory markers (*CXCL12* and *TNFSF9*) or pro-inflammatory markers (*TNF-α* and *IL32*) showed no significant changes. However, the collagen-treated groups showed reduced *IL6* expression levels compared with the control group, which possibly facilitated the M2 polarization of macrophages. The effects of different inflammatory factors on macrophage phenotypes were similar, distinct, or even opposing. Thus, macrophage polarization influenced by MSC-derived paracrine secretions was likely shaped by the interplay of multiple factors. These findings suggested that paracrine secretions from collagen-stimulated MSCs possibly modified the macrophage functional states, potentially affecting inflammation resolution or activation during tissue repair.

## Discussion

In most tissue engineering studies, scaffolds are generally regarded as physical structures providing mechanical support and surfaces for cell attachment and proliferation, with their bioactivity primarily dependent on the incorporation of exogenous biological molecules [54]. In contrast to the extensive research on biophysical cues of biomaterials, our study delves into the biochemical properties of scaffolds and demonstrates that recombinant collagens can inherently deliver biochemical signals capable of modulating the secretome of mesenchymal stem cells (MSCs). These biochemical cues may not be strictly limited to downstream pathways of integrin signaling, and the underlying mechanisms merit further investigation.

In our study, we employed synthetic biology approaches to produce and characterize recombinant type I and type III collagens, in a manner analogous to how the effects of drugs or chemicals are studied. While native collagen possesses a classical triple-helical structure, the recombinant collagens used in our work lack a fully preserved triple helix. Nevertheless, increasing evidence indicates that critical functional motifs embedded in collagen sequences—such as GER [55,56], GFPGER [57,58], and KPGPRGQRGPTGPRGE [59] in type I and III collagens—retain bioactivity and can regulate cell adhesion, migration, and intracellular signaling via integrin interactions. Other sequences, including GAPGAPGSQGAPGLQ [60,61], have also been shown to support cell adhesion and matrix remodeling [[62], [63], [64]]. Moreover, composite domains in type III collagen—such as GERGAPGFRGPAGPNGIPGEKGPAGERGAP [[65], [66], [67]]—have been reported to enhance regenerative microenvironments and modulate immune responses [[68], [69], [70], [71], [72]]. Collectively, these findings support our observation that recombinant collagens, despite their structural differences from native collagens, retain sufficient biochemical information to functionally interact with MSCs.

Notably, recombinant type I and type III collagens may primarily interact with α1β1, α2β1, and α11β1 integrins, which are known to mediate collagen-dependent cell adhesion and cytoskeletal remodeling [29,73]. We further observed that different types of collagen exhibited distinct effects on MSC signaling pathways. Recombinant type I collagen primarily activated the *FAK/RHOA/ROCK* axis, suggesting its regulatory role in cytoskeletal remodeling and cell adhesion, whereas recombinant type III collagen markedly activated the *PI3K/Akt* pathway, which may be associated with its function in promoting cell survival, proliferation, and anti-apoptotic responses. In contrast, bovine type I collagen mainly influenced glycolytic metabolism, indicating a specific role in regulating cellular energy metabolism. Furthermore, we integrated GO and KEGG enrichment results to construct a functional interaction network (Fig. S20), revealing potential connections among *PI3K/AKT*, *HIF-1α*, glycolysis, and other enriched pathways. Although all collagen types interact with MSCs via integrins to trigger downstream signaling, the preferentially activated pathways differ, which may underlie their distinct biological effects on regulating cell fate and function.

While in vitro experiments provide a controlled environment to elucidate the molecular interactions between recombinant collagen and mesenchymal stem cells (MSCs), they do not fully replicate the complexity of the in vivo tissue microenvironment. MSC behavior in vivo is influenced by dynamic factors, such as mechanical factors, immune cell interactions, and matrix remodeling processes, which cannot be fully captured in in vitro experiments. This is a limitation of the current study and warrants further studies involving in vivo models to validate the observed mechanisms and evaluate the therapeutic potential and safety of recombinant collagen-based materials in physiological settings.

In addition, slight degradation of recombinant collagen was observed after 72 h of co-culture with MSCs（Fig. S21）, but it remains unclear whether the triple-helical structure or molecular weight (Mw) influences its bioactivity. In our previous work, we constructed a recombinant type I collagen (PF-I-80) using synthetic biology, based on residues 242–1147 of the human collagen type I α1 chain. This sequence retained key bioactive sites such as GPP and GER and exhibited comparable biological efficacy to native type I collagen α1 chains [74]. These findings indicate that studying the biological functions of various collagen-derived sequences may reveal whether enhanced stress responses can improve stem cell differentiation potential—an intriguing direction for future research.

Future studies should also determine the optimal concentration of recombinant collagens to support long-term culture and investigate dose- and time-dependent effects. This research will expand to a broader range of cell types to evaluate the generalizability of sequence-specific effects on human MSCs. Moreover, the potential synergistic effects of combining different recombinant collagen types warrants exploration.

The omics technologies applied in our study provided comprehensive insights into cell responses, significantly improving our understanding of the regenerative potential of specific bioactive materials. Based on these findings, we advocate for the development of recombinant collagen-based biomaterials targeted to the site of tissue repair. This strategy offers a concentrated and efficient pathway for tissue regeneration, potentially enhancing both the repair process and functional recovery outcomes.

Therefore, future studies should utilize multi-omics approaches to elucidate the relationship between recombinant collagen structure, molecular weight, and bioactivity, to achieve a more comprehensive understanding of its biochemical regulatory mechanisms.

## Conclusions

In this study, recombinant type I and III collagens were produced using synthetic biology, and their biochemical interactions with mesenchymal stem cells (MSCs), along with the resulting biological processes, were systematically elucidated through multi-omics analyses. Integrated transcriptomic and proteomic analyses revealed that all three collagens—recombinant type I, recombinant type III, and bovine type I—induce broad but distinct cellular responses, primarily mediated via integrins. Specifically, recombinant type I collagen modulates amino acid metabolism through the *FAK/RHOA/ROCK* signaling axis, enhancing MSC paracrine functions related to ECM deposition, angiogenesis, and immune regulation. Recombinant type III collagen primarily regulates MSC paracrine activity via activation of the *PI3K/AKT* pathway, whereas bovine type I collagen promotes glycolysis through *HIF-1α*, further supporting paracrine functions in angiogenesis and immune modulation.

Recombinant collagen-stimulated MSC paracrine activity significantly influences the behavior of diverse cell types, highlighting its potential as a tissue-regenerative scaffold material. This study provides the first comprehensive multi-omics insight into recombinant collagen–MSC interactions, offering direct guidance for the development of recombinant collagen-based regenerative scaffolds. Moreover, it underscores the utility of omics approaches for high-throughput, multi-level evaluation of cell–biomaterial interactions, informing future selection and design of tissue engineering scaffolds.

## Experimental methods

### Strain construction, protein expression, and purification

The genes encoding type I and type III collagens were integrated into the pPICZαA plasmid (Invitrogen, Waltham, USA) at the Xho I and Not I restriction sites, generating recombinant plasmids pPICZαA-COLI and pPICZαA-COLIII, respectively. These plasmids were transformed into *Escherichia coli* DH5α (TaKaRa, Dalian, China) via heat shock. The resulting strains were cultured overnight at 37 °C on Luria-Bertani agar plates supplemented with 100 μg/mL of zeocin (Sigma-Aldrich, St. Louis, USA). The recombinant plasmids were extracted from the bacterial cultures using a plasmid extraction kit (Tiangen Biochemical Technology, Beijing, China).

Subsequently, the plasmids were introduced into the *Pichia pastoris* cells (Invitrogen, Waltham, USA) via electroporation. The transformed yeast cells were then plated on YPD agar plates supplemented with 100 μg/mL of zeocin and incubated at 30 °C for 2–3 days. The positive colonies were then transferred to BMGY medium (Sangon, Shanghai, China) and cultured at 30 °C on a rotary shaker at 250 rpm until the OD600 value reached 2–8, at which point the cells were harvested by centrifugation. The cell pellet was resuspended in BMMY medium to an OD600 value of 1.0 for the induction of recombinant collagen expression. The induction was performed in a 5 L fermenter, with 1 % methanol (Sigma-Aldrich, Sigma-Aldrich, St. Louis, USA) added every 24 h for 72 h to induce collagen gene expression. The culture was then scaled up in the BMMY medium to enhance protein yield.

The culture medium was sterilized, and the temperature and pH were set to 30 °C and 5.0, respectively. A 10 % inoculum of seed solution was used to inoculate the fermenter, and the agitation speed was gradually increased until the dissolved oxygen (DO) level reached approximately 70–80 %. Glycerol was added in pulses of 10–12 s every minute until the DO level reached 100 %, after which a 1 h starvation period was imposed. Methanol supplementation was then initiated with pulses of 2–3 s every minute. Once the DO levels stabilized, the supplementation rate was increased accordingly, and the methanol induction phase lasted approximately 40 h. The OD values were measured every 2 h throughout the fermentation process.

Post-fermentation, the culture broth was centrifuged at 8000 rpm for 20 min. The supernatant was then further processed using a hollow fiber column (Sigma-Aldrich, St. Louis, USA) to remove any impurities and concentrated via ultrafiltration using a 100 kDa cutoff membrane (Sigma-Aldrich, St. Louis, USA). The collagen proteins were then purified using MMC cation exchange chromatography (Cytiva, Hong Kong). To ensure high purity, the proteins were desalted through ultrafiltration, followed by lyophilization to obtain the proteins in a dry form.

### Protein structure characterization

The processed collagen samples were subjected to enzymatic digestion using trypsin & Glu-C, elastase, and trypsin & chymotrypsin. The digested samples were then analyzed using LC-MS/MS to identify the protein structures (BTParker, Beijing, China).

The lyophilized protein samples were dissolved in deionized water to a final concentration of 1 mg/mL, and the samples were then subjected to UV–Vis spectrophotometry to determine the primary characteristic absorption peaks. Circular dichroism (CD) spectroscopy was performed on a 0.1 mg/mL protein solution, with the CD values recorded over a wavelength range of 180–280 nm to assess the secondary structure content. Additionally, Fourier-transform infrared spectroscopy was conducted, and the infrared spectra of the samples were recorded in the wavelength range of 500–4000 cm^−1^ to further analyze the structural features of the protein.

### Cell culture

All cell types were purchased from Shanghai Fuheng Biotechnology Co. Ltd. and cultured under a humidified atmosphere containing 5 % CO_2_ at 37 °C. According to the supplier, BMSCs were stained for purity by flow cytometry (BD Biosciences) using PE anti-human CD90, (BioLegend, 328109), APC anti-human CD73, (BioLegend, 344005), PE anti-human CD105, (BioLegend, 323205), APC anti-human CD45, (BioLegend, 982304), and PE anti-human CD34, (BioLegend, 343505). The analysis was performed with three biological replicates.The MSCs used in this study were negative for CD34 and CD45 and positive for CD90, CD73, and CD105.

The MSCs were cultured in MSCM medium (ScienCell Research Laboratories, California, USA), and the MSCs between passages 3 and 6 were used for subsequent experiments. HSFs were cultured in DMEM supplemented with 10 % fetal bovine serum (FBS) (Gibco, Waltham, USA) and 1 % penicillin/streptomycin (P/S) (Sigma-Aldrich, St. Louis, USA). Human umbilical vein endothelial cells (HUVECs) were maintained in ECM medium (ScienCell Research Laboratories, California, USA). Human THP-1 cells were cultured in THP-1 monoclonal growth medium (YM-SD-011, ubigene, China). The THP-1 cells were differentiated into M1 macrophages through treatment with 300 ng/mL phorbol 12-myristate 13-acetate (PMA, Sigma-Aldrich, St. Louis, USA) and 100 ng/mL lipopolysaccharide (LPS, Sigma-Aldrich, St. Louis, USA). Differentiation into M2 macrophages was induced using 100 ng/mL IL-4 (Sigma-Aldrich, St. Louis, USA). The culture media were refreshed every 2 days.

To collect the paracrine substances (PSs), the MSCs were treated with or without 1 mg/mL of type I, type III, or bovine type I collagen in the serum-free medium. After 24 h of culture, the supernatant was collected and centrifuged at 2000 rpm for 10 min to remove any dead cells and cellular debris. The centrifuged supernatant was then collected and stored at 4 °C until further use. For subsequent cell culture experiments, the PS was mixed with complete growth medium at a 1:1 ratio.

### Cell proliferation and metabolism

To assess the effects of recombinant type I and type III collagens on cell proliferation, cells were seeded at a density of 10^4^ cells/well into 96-well plates. The cells were then treated with various concentrations of type I and type III collagens for 24 h to determine the optimal concentration for stimulating cell proliferation. Bovine type I collagen (approved by the China Food and Drug Administration) was used as the control. Fibroblasts or HUVECs were then cultured with conditioned media from MSCs treated with 1 mg/mL of type I, type III, or bovine type I collagen. At specified time points, 10 % (v/v) CCK-8 solution was added to replace the culture medium. After incubating at 37 °C for 1 h, the optical density of the solution was measured at 450 nm using a microplate reader (BioTek, Santa Clara, USA).

For cell viability assessment, 1 × 10^5^ cells were seeded into six-well plates and cultured for 24 h in fresh medium with and without 1 mg/mL collagen. The cells were then stained using a Calcein-AM/PI double staining kit (CA1630, Solarbio, Beijing, China), according to the manufacturer’s instructions. After 20 min of incubation with the staining solution, the live and dead cells were observed under a fluorescence microscope (Nikon), and images were captured from the same field of view.

The MSCs (1 × 10^4^) were seeded in 96-well plates and cultured for 24 h in fresh medium with and without 1 mg/mL of type I, type III, or bovine type I collagen to evaluate their metabolic activity. At specified time points, the medium was replaced with growth medium containing 10 % (v/v) Alamar Blue solution. After 2 h of incubation, the fluorescence intensity was measured at 590 nm with an excitation wavelength of 560 nm using a microplate reader (BioTek, Santa Clara, USA).

### Cell migration

For cell migration assays, serum-free medium containing collagen was placed in the lower chamber, while BMSCs, fibroblasts, or HUVECs were seeded in the upper chamber (8 μm pores, Corning). The upper chamber was filled with serum-free medium. After 24 h of culture, the chambers were collected and the cells on the membrane were fixed with 4 % paraformaldehyde. The cells were then stained with 0.5 % crystal violet (Maclin) and observed with a bright-field microscope.

### Osteogenic, chondrogenic, and adipogenic differentiation

To induce osteogenic differentiation, the MSCs were cultured in osteogenic differentiation medium (ScienCell Research Laboratories, California, USA) containing 10 % fetal bovine serum (FBS), 1 % P/S, 10 mM β-glycerophosphate, 100 nM dexamethasone, and 50 µg/mL ascorbic acid for 21 days. Osteogenic differentiation was then assessed by staining with Alizarin Red S to visualize the mineralized nodules, which were quantified by dissolving the dye with 10 % cetylpyridinium chloride and measuring the absorbance at 570 nm.

For chondrogenic differentiation, the MSCs were cultured in chondrogenic differentiation medium (ScienCell Research Laboratories, California, USA) supplemented with 10 % FBS, 1 % P/S, and 50 µg/mL ascorbic acid in 15 mL conical tubes for 21 days. The formation of the cartilage matrix was evaluated by staining with Alcian Blue, and the extent of matrix formation was quantified by measuring the dye at 620 nm.

To induce adipogenic differentiation, the MSCs were cultured in adipogenic differentiation medium (ScienCell Research Laboratories, #7501) containing 10 % FBS, 1 % P/S, 1 µM of dexamethasone, 0.5 mM isobutylmethylxanthine, and 10 µg/mL of insulin for 21 days. Adipogenic differentiation was then assessed by staining with Oil Red O to visualize the lipid droplets. The amount of lipid accumulation was quantified by extracting the dye with isopropanol and measuring the absorbance at 500 nm.

### F-actin staining

F-actin staining was performed to observe the cell morphology and adhesion. The MSCs were seeded at a density of 1 × 10^4^ cells per dish on laser scanning confocal plates (20 mm, Nest, Wuxi, China) and cultured in fresh medium with and without 1 mg/mL of recombinant type I collagen, recombinant type III collagen, or bovine type I collagen for 7 days. On day 7, the cells were fixed in 4 % paraformaldehyde for 15 min. To enhance cell permeability, the samples were incubated in a permeabilization buffer (Beyotime, Beijing, China) for 15 min.

Subsequently, the cells were incubated with Actin-Tracker Green-488 (Beyotime, Beijing, China) working solution for 1 h to label F-actin. The nuclei were stained with 10 mg/mL of DAPI (C0065, Solarbio, Beijing, China) for 5 min. The stained samples were then observed and imaged using a laser scanning confocal microscope (Nikon, Japan).

To analyze F-actin polymerization, the fluorescence images were imported into ImageJ software for integrated intensity measurement. The cell perimeter and interior regions were determined as described in the literature [75]. To visualize the orientation distribution of F-actin, the Orientation J plugin was used [76]. Quantitative orientation measurements were performed by specifying regions of interest, with the cell axis direction defined as 0°.

### RNA extraction and qPCR

Total RNA was extracted from cultured cells using a Total RNA Extraction Kit (Genesand, Beijing, China). Subsequently, cDNA was synthesized using a cDNA Reverse Transcription Kit (Thermo, Waltham, USA). Gene expression was assessed using specific primers (Qingke Biotechnology, Beijing, China) and SYBR Green (Roche, Sigma-Aldrich, St. Louis, USA) via real-time PCR.

The primers used in this study are listed in Table S1, with GAPDH selected as the housekeeping gene. The results are presented as the ratio of target gene expression to the housekeeping gene, normalized to the control group.

### RNA sequencing and bioinformatics analysis

The MSCs were cultured in growth media with and without 1 mg/mL of type I collagen, type III collagen, or bovine type I collagen. After 3 days of culture, the cells were collected and analyzed using a high-output HiSeq platform.

Gene expression was determined by calculating the FPKM for uniquely mapped reads overlapping coding exons, with gene length normalization. The transcriptome data were analyzed according to the BGI Omics analysis website (https://biosys.bgi.com/), OmicStudio (https://www.OmicStudio.cn/), and REVIGO website (https://REVIGO.irb.hr/).

Gene expression values were normalized to z-scores for hierarchical data clustering and heatmap generation. The DEGs were identified through pairwise comparisons with a significance threshold of p < 0.05. GO term clustering was visualized using REVIGO, and protein–protein interaction networks were constructed using the STRING database and Cytoscape.

### Proteomics analysis of the MSC secretome

The MSCs were cultured in growth media with and without 1 mg/mL of type I collagen, type III collagen, or bovine type I collagen. After 3 days of culture, the cell supernatants were collected and centrifuged at 2000 rpm to remove any dead cells and cell debris. The secretome from the MSCs was analyzed using a proteomics approach based on data-independent acquisition technology.

Proteomics data were analyzed using the DAVID website (https://DAVID.ncifcrf.gov/) and OmicStudio tools (https://www.OmicStudio.cn/). The protein expression values were normalized to z-scores for hierarchical clustering and heatmap generation. DEPs were identified through pairwise comparisons among the three groups, and DEPs with a p-value < 0.05 were selected for GO and KEGG enrichment analyses.

### ELISA analysis of secreted factors

The MSCs were seeded into six-well plates at a density of 2 × 10^5^ cells per well and cultured with and without 1 mg/mL of type I collagen, type III collagen, or bovine type I collagen for 3 days. After cultivation, the MSC supernatants were collected and centrifuged at 2000 rpm for 10 min to remove any cell debris.

The levels of VEGFA (Shanghai Enzyme-Linked Biotechnology, ml1057663), IGF1 (Shanghai Enzyme-Linked Biotechnology, ml060962), TNF-α (Shanghai Enzyme-Linked Biotechnology, ml077385), and IL-10 (Shanghai Enzyme-Linked Biotechnology, ml064299) in the supernatants were measured using ELISA, according to the manufacturer's instructions.

### Data analysis

Statistical significance among multiple groups was determined using one-way ANOVA with Tukey's post hoc test. Unless otherwise specified, a p-value < 0.05 was considered statistically significant. All analyses were conducted using GraphPad Prism software.

## Author statement

Taishan Liu, LinLin Qu, Huan Lei and Daidi Fan participated in the study design. Taishan Liu performed the experiments and statistical analyses and wrote the first draft of the manuscript. Juanli Dang, Chenhui Zhu, Xiaoxuan MA were involved in changing the content of the manuscript. Daidi Fan was mainly responsible for the final content. All authors read and approved the final manuscript.

## Availability of data and materials

All data and materials are available to the researchers once published.

## Compliance with ethics requirements

This article does not contain any studies with human or animal subjects.

## Declaration of competing interest

The authors declare that they have no known competing financial interests or personal relationships that could have appeared to influence the work reported in this paper.
