# Supplementary material for: Multi-omics elucidation of recombinant collagen‐mediated modulation of mesenchymal stem cell functions
Source: J Adv Res. 2025 Sep 17;84:305–21. doi: 10.1016/j.jare.2025.09.032 (PMC13227244; doi:10.1016/j.jare.2025.09.032)
Supplement: Supplementary Data 1 [file mmc1.docx]

Supplementary Information

Multi-Omics Elucidation of Recombinant Collagen‐Mediated Modulation of Mesenchymal Stem Cell Functions


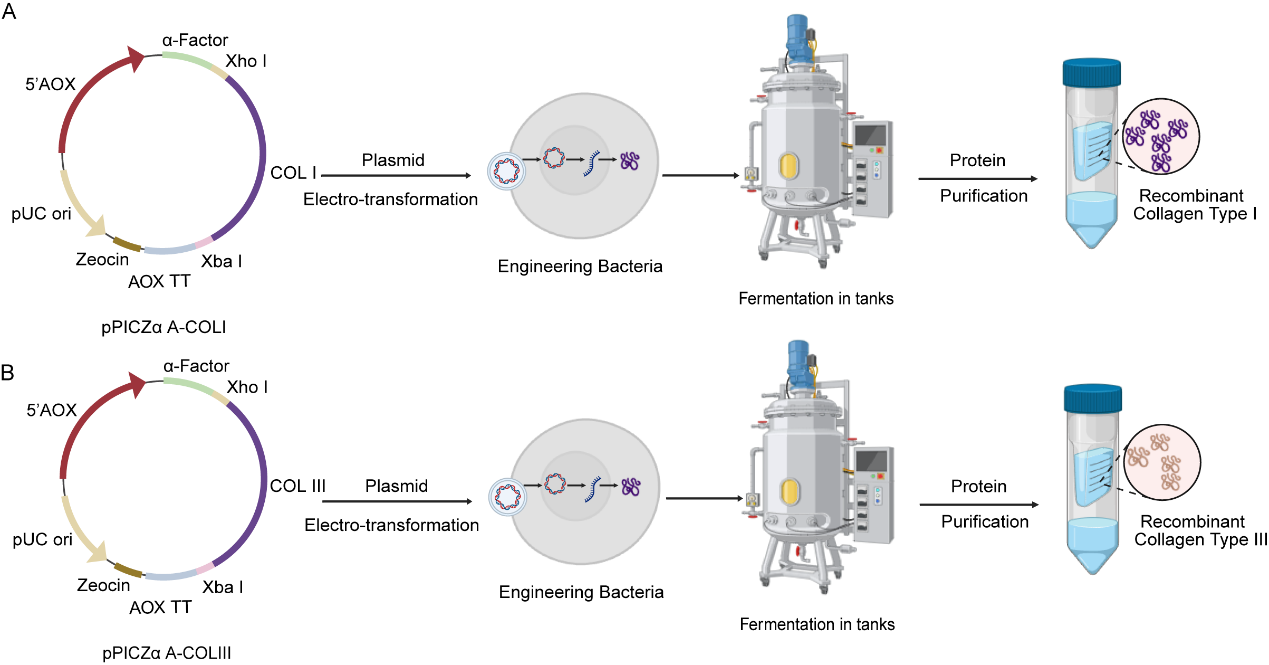


Figure S1. Schematic representation of collagen synthesis


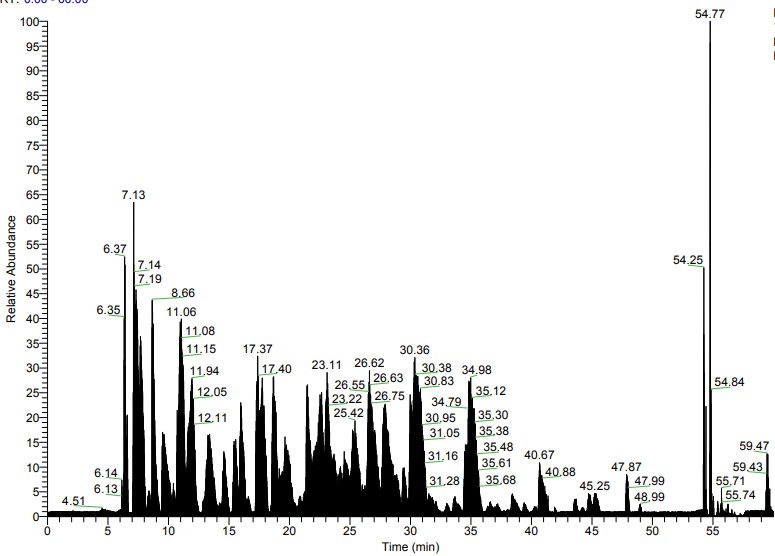

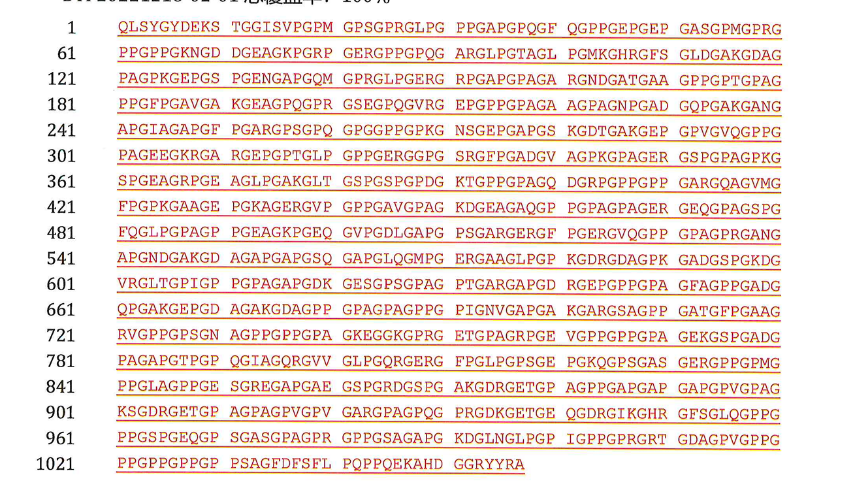


Figure S2. Type I collagen mass spectral information
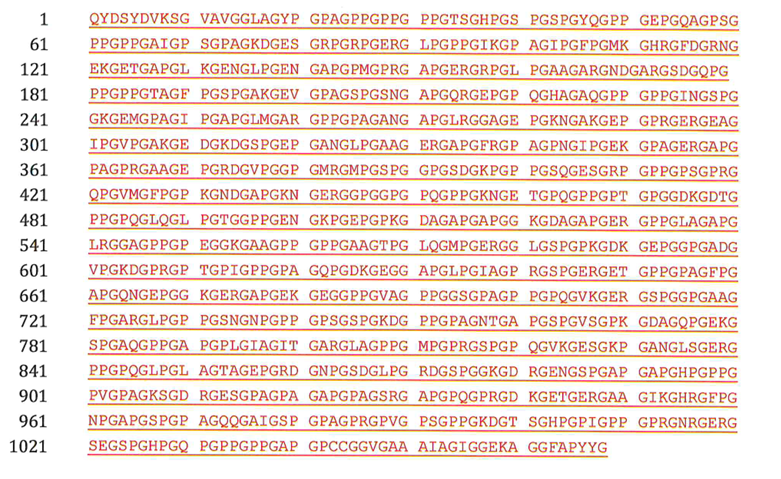


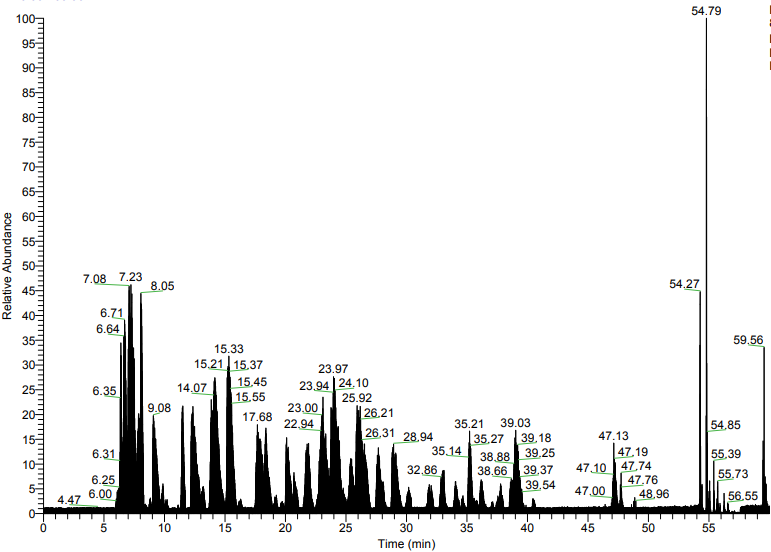
Figure S3. Type III collagen mass spectrometry information
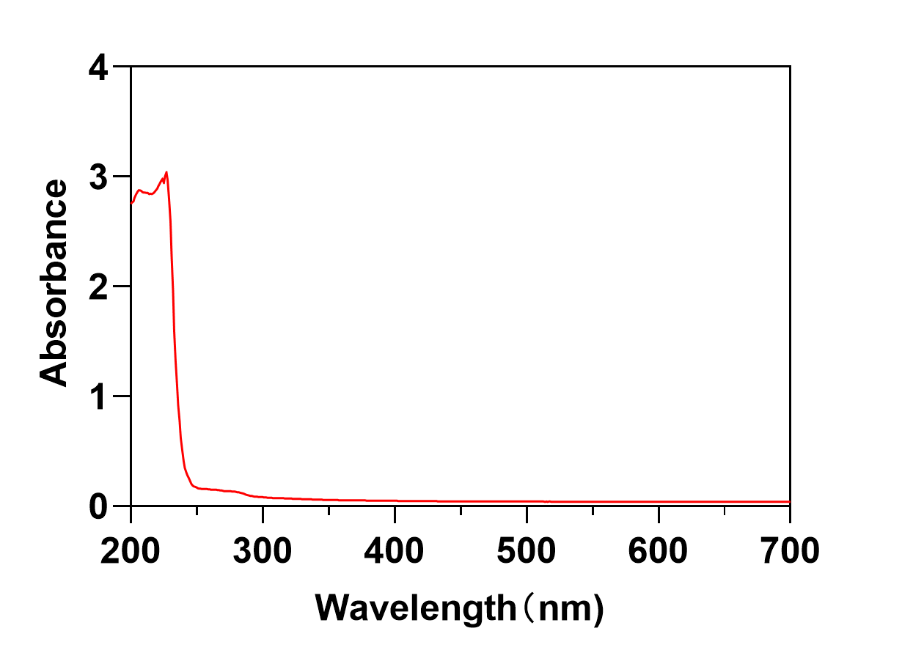


Figure S4.UV absorption spectrum of type I collagen
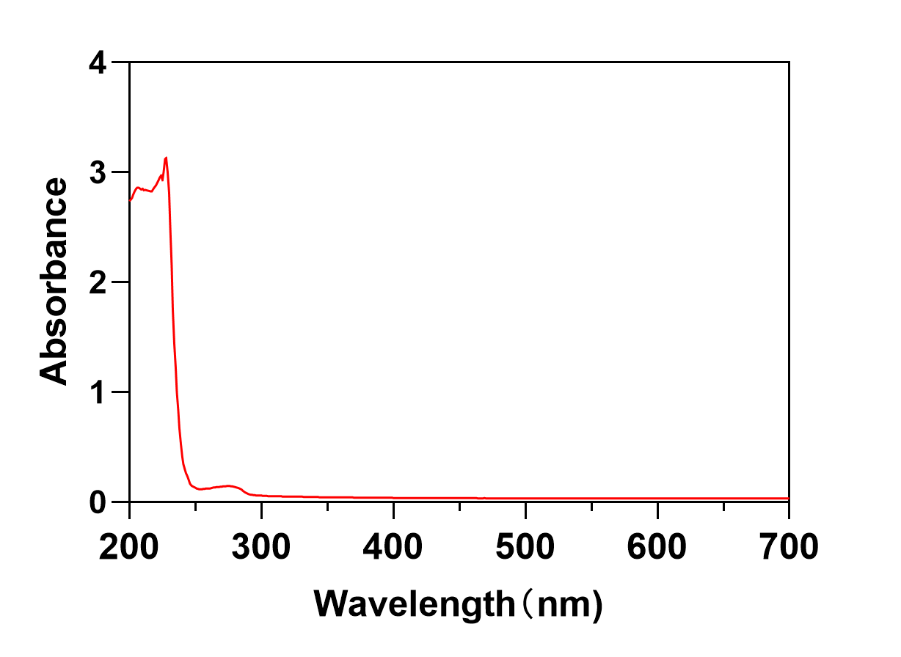


Figure S5.UV absorption spectrum of type III collagen


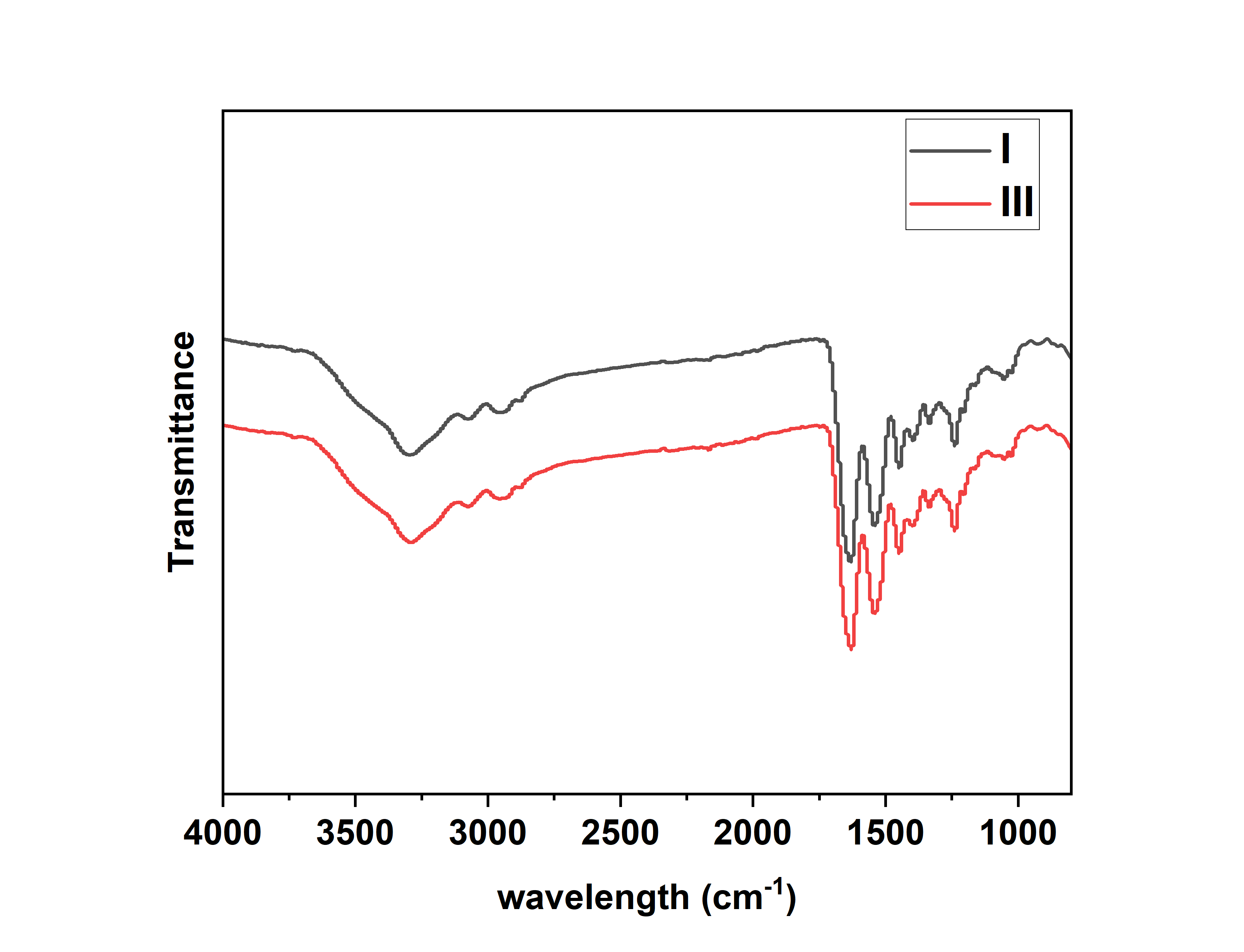


Figure S6. recombinant type I and type III Fourier infrared spectra


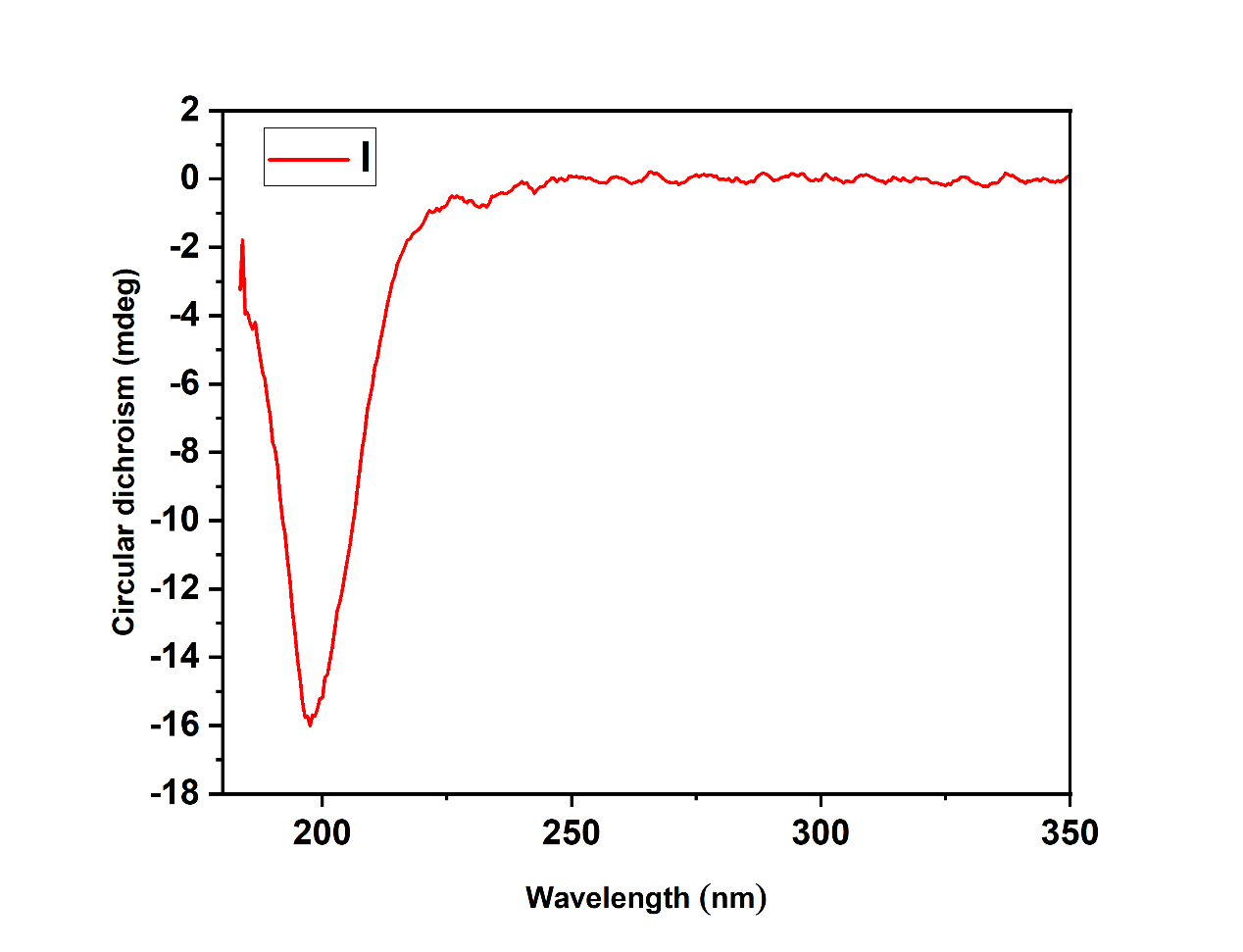


Figure S7.UV absorption spectrum of recombinant type I collagen


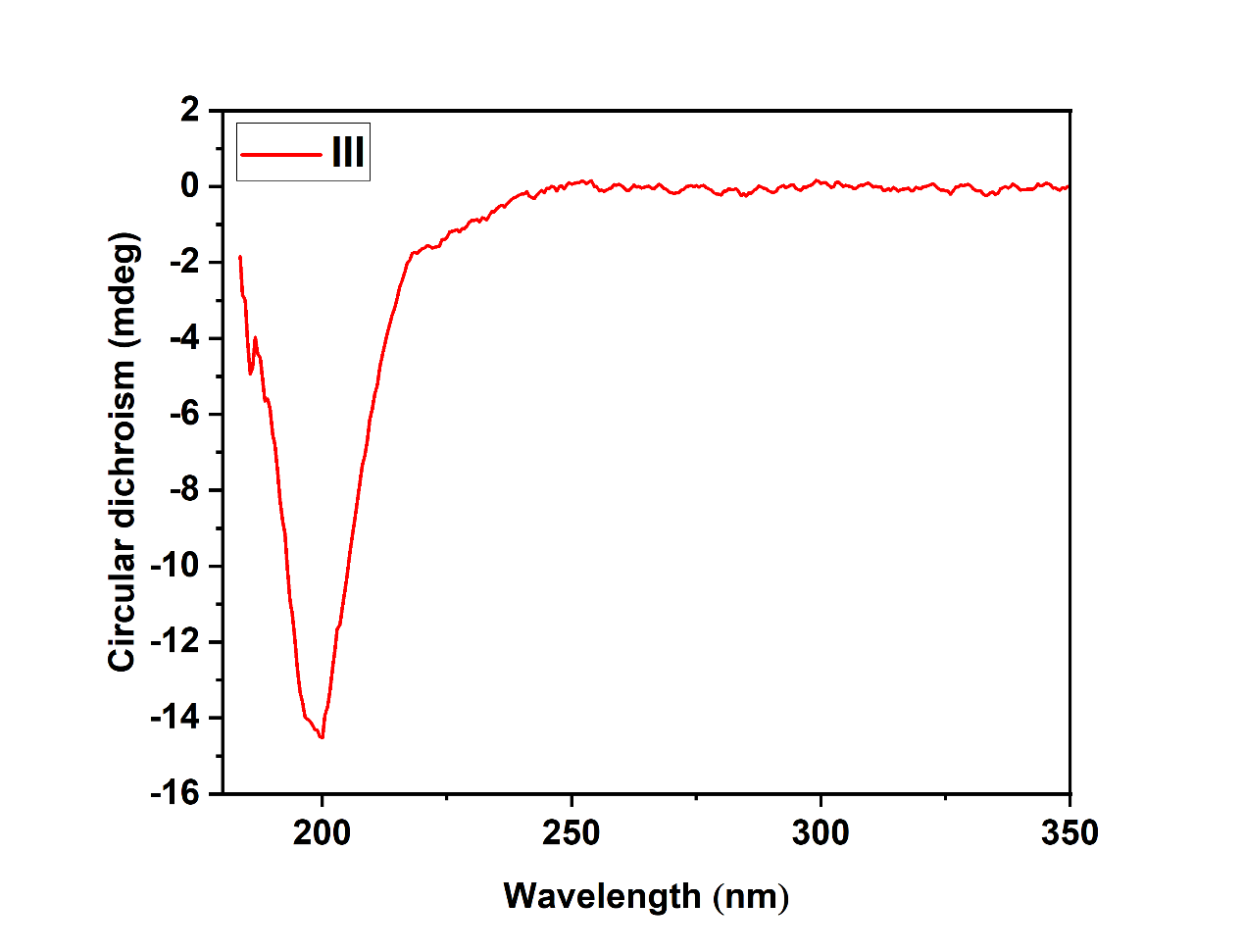


Figure S8.UV absorption spectrum of recombinant type III collagen


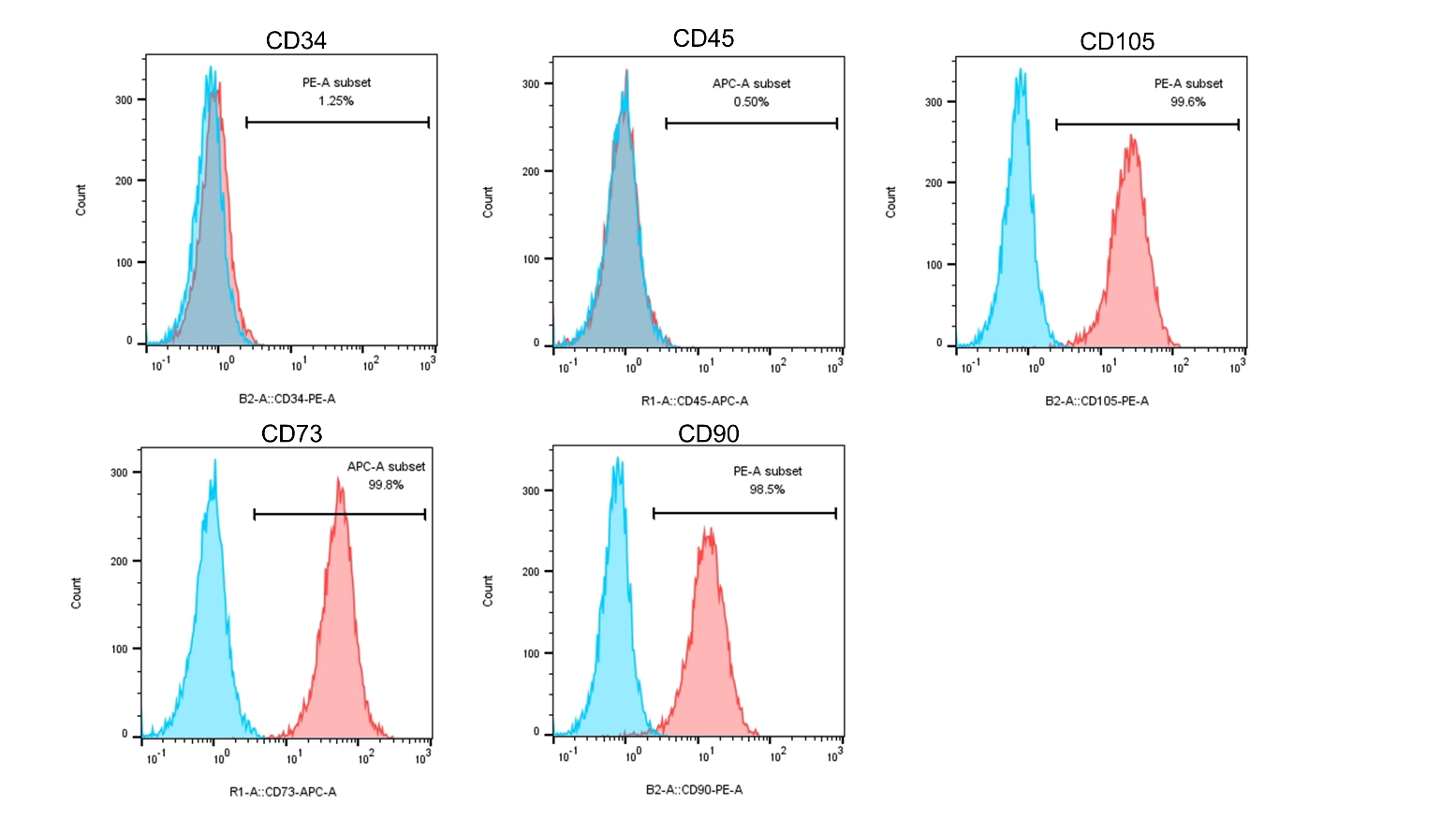


Figure S9. Flow cytometry was used to measure the mesenchymal stem cell markers CD34, CD45, CD105, CD73, and CD90 (n=3).


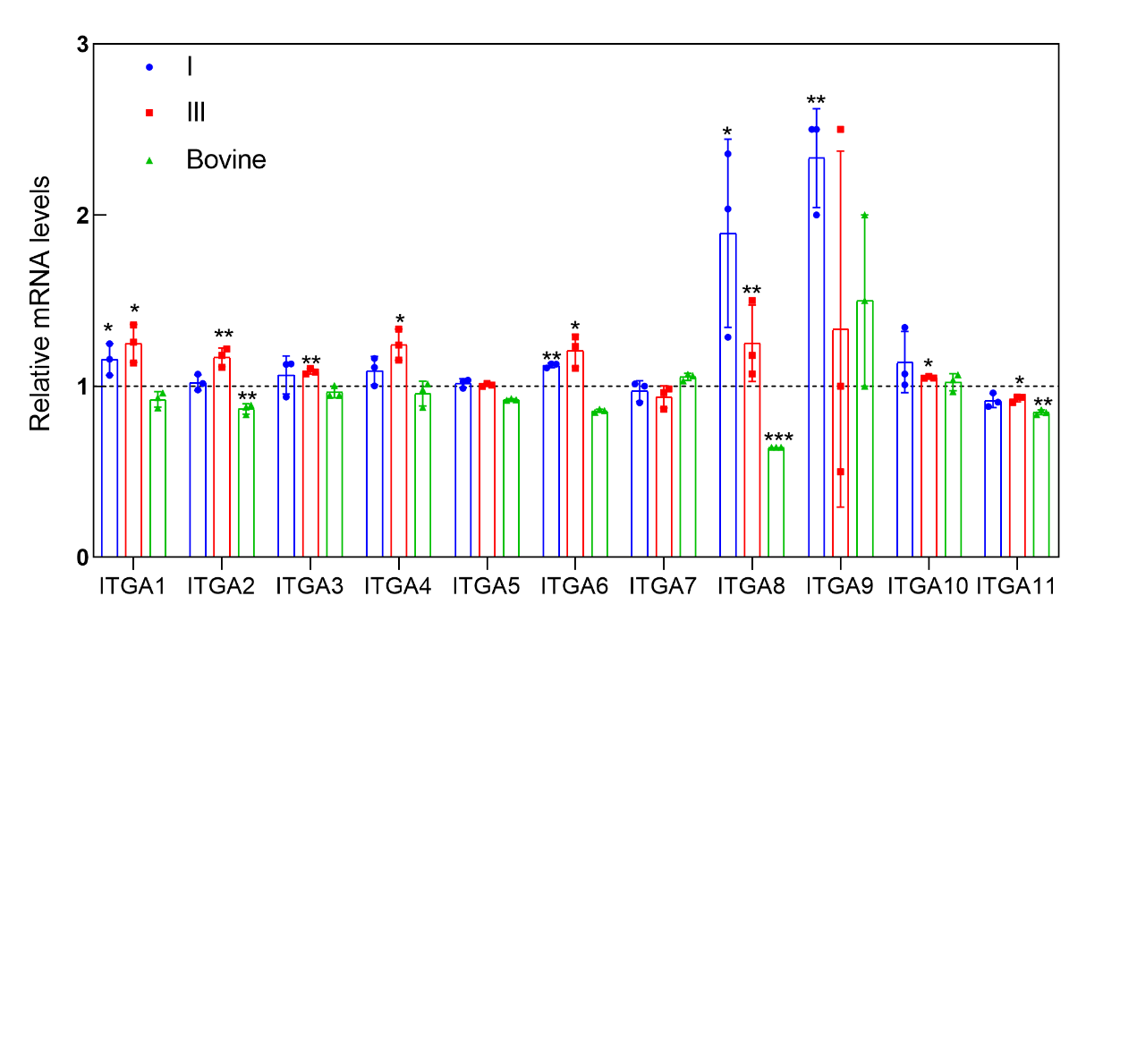


Figure S10. mesenchymal stem cell integrin A expression. (n=3) Significant difference (one-way ANOVA): *P < 0.05, **P < 0.01, and ***P < 0.001.


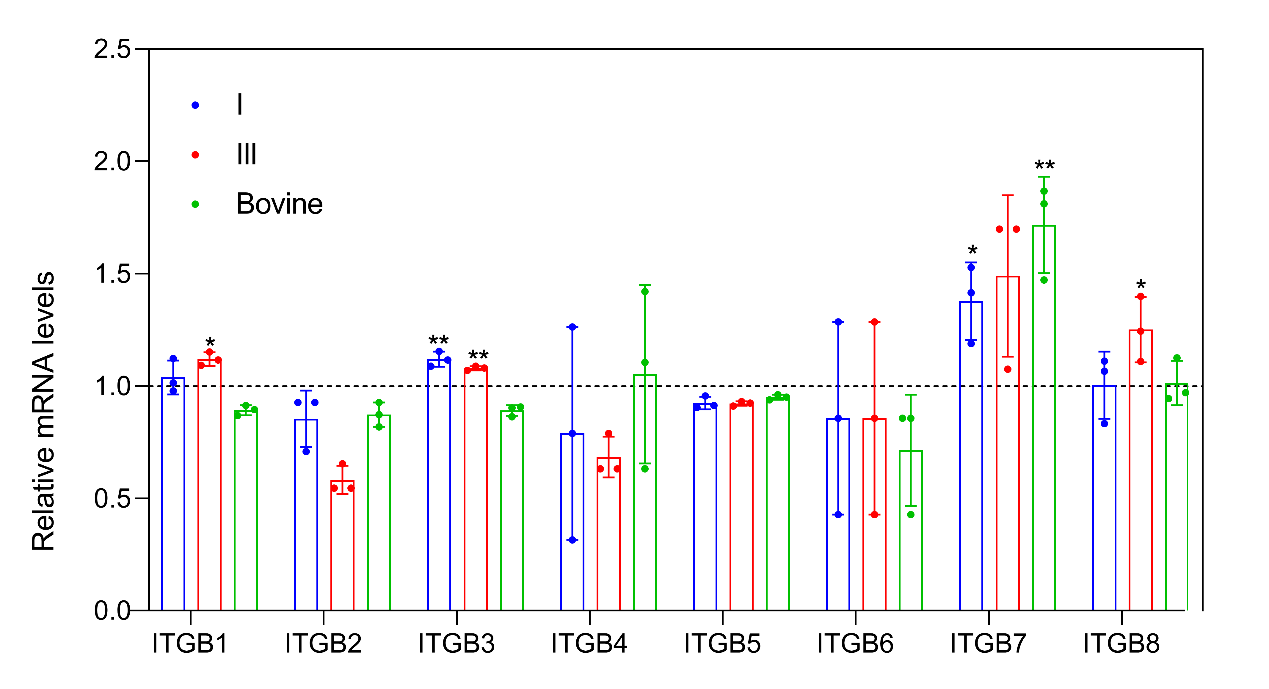


Figure S11. mesenchymal stem cell integrin B expression. (n=3) Significant difference (one-way ANOVA): *P < 0.05, **P < 0.01, and ***P < 0.001.


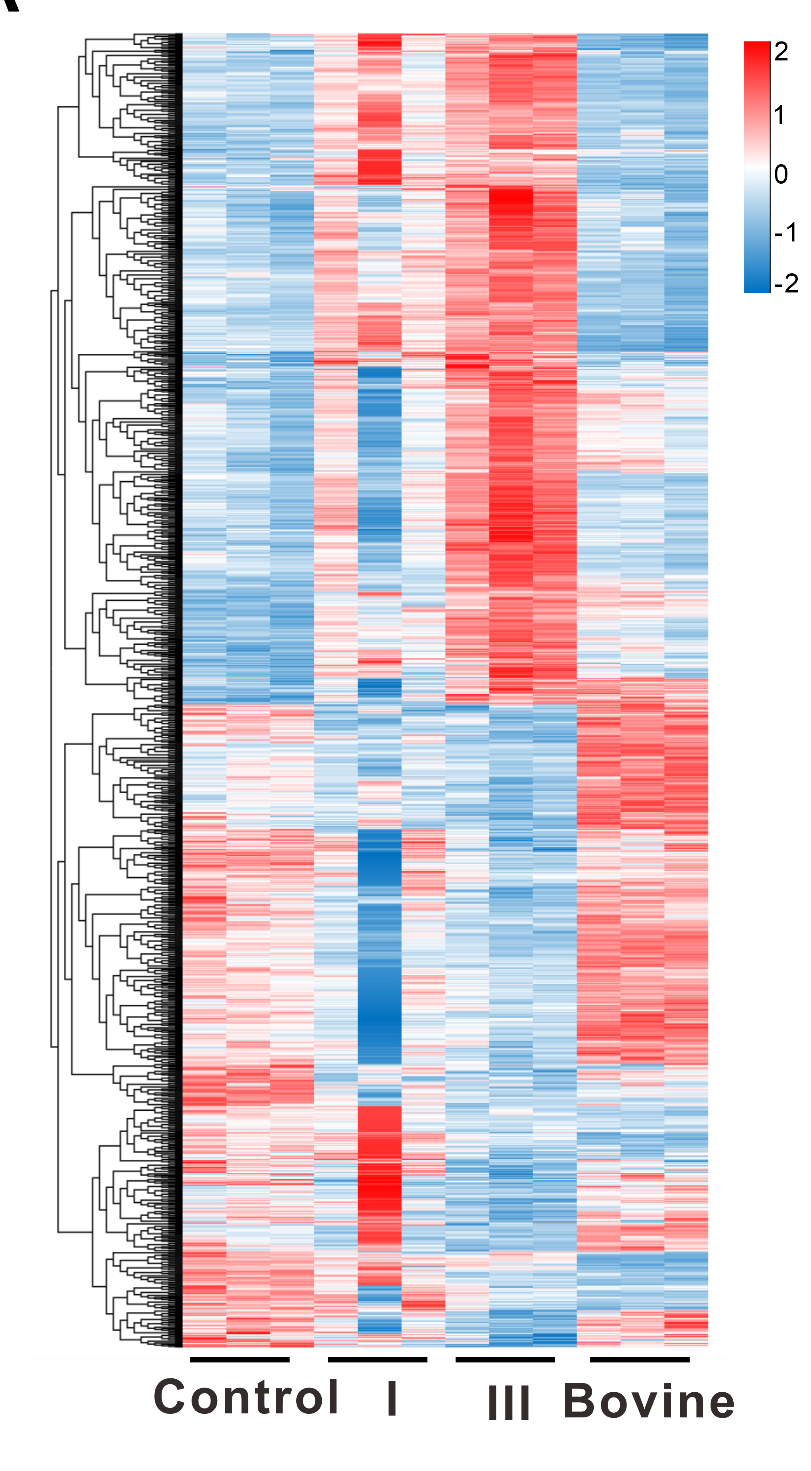


Figure S12 .Heatmap of gene expression levels after hierarchical cluster analysis (p < 0.05)


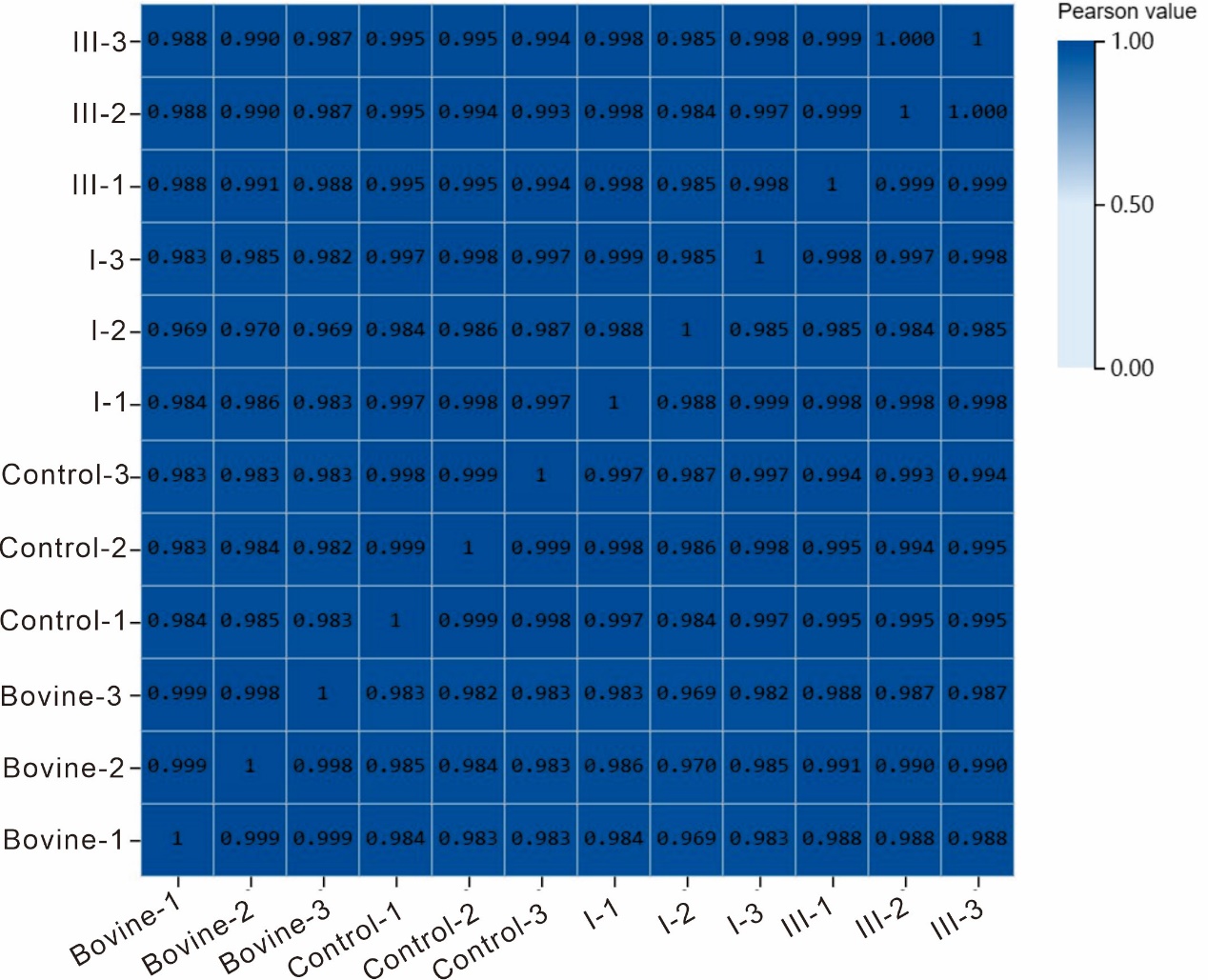


Figure S13. correlation heatmap showing the relationship between duplicate samples from Control group, collagen type I group, collagen type III group and cowhide collagen type I group. Correlation coefficients (R = 0-1) indicate the strength of correlation across samples.


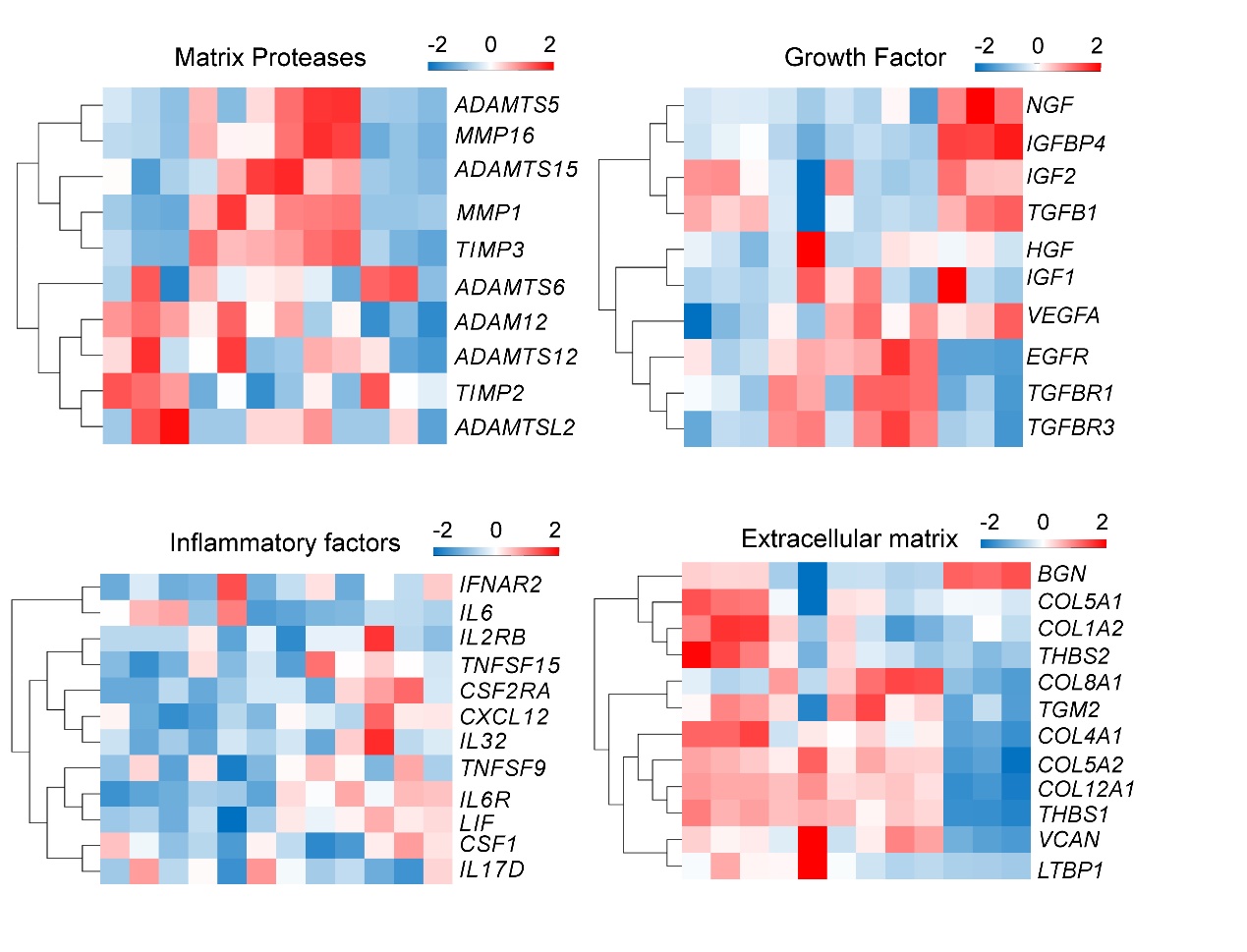
 Figure S14, A) Heat map of DEGs associated with matrix proteases, B) Heat map of DEGs associated with growth factors, C) Inflammatory factors, and D) Extracellular matrix (ECM). Results are expressed as mean ± SD


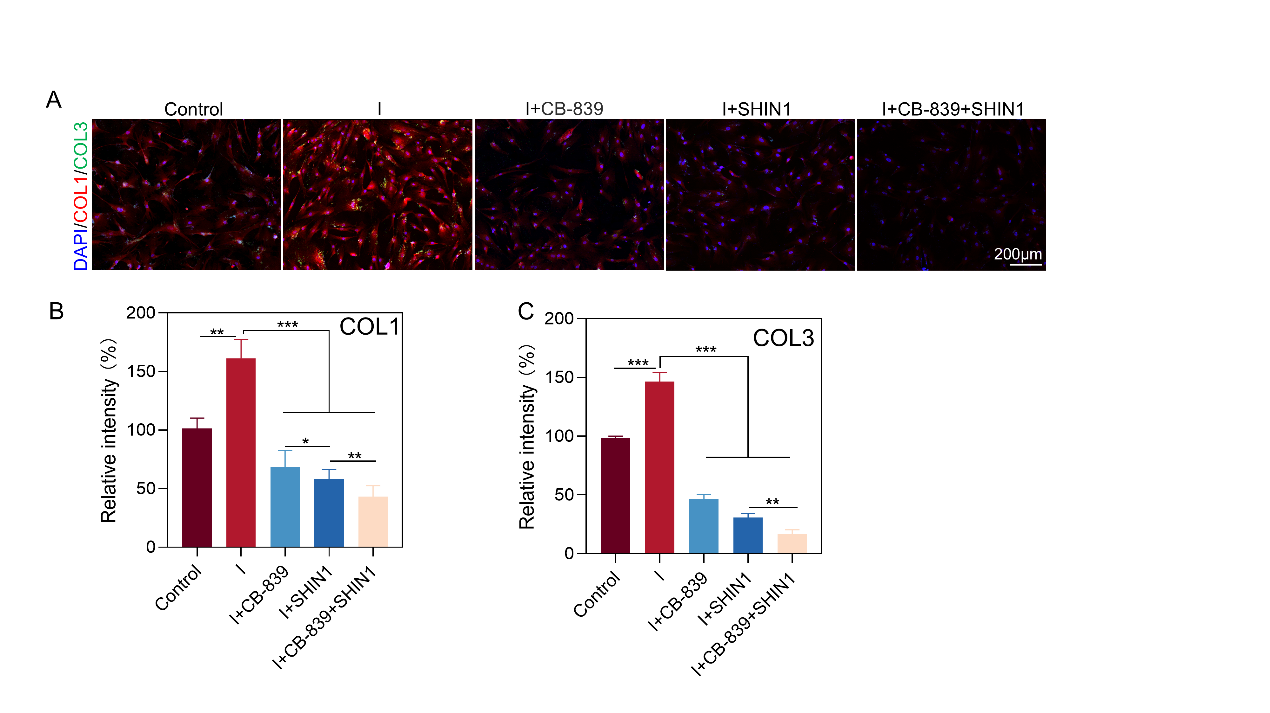


Figure S15. (A) Immunofluorescence staining of type I collagen (COL1) and type III collagen (COL3) in MSCs after treatment with recombinant type I collagen.(B–C) Quantitative fluorescence intensity analysis of COL1 and COL3(n=3). Results are expressed as mean ± SD. Significant difference (one-way ANOVA): *P < 0.05, **P < 0.01, and ***P < 0.001.


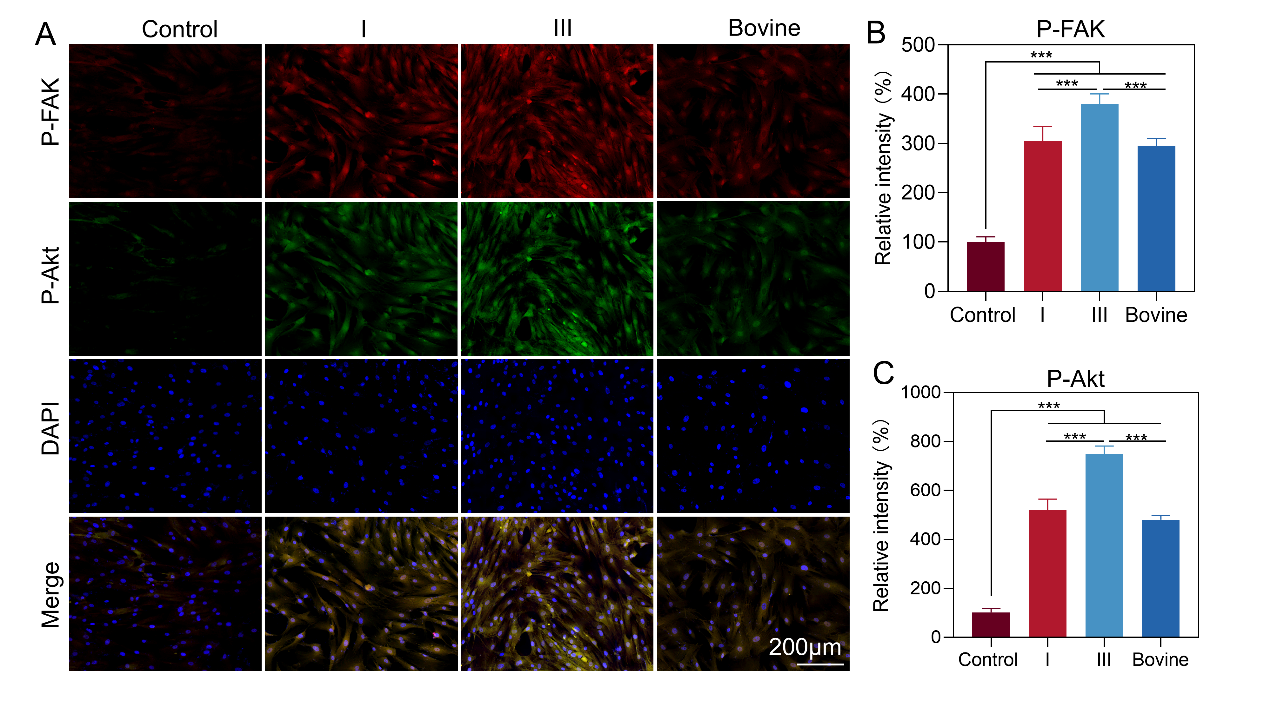


Figure S16. (A) Immunofluorescence staining of phosphorylated FAK and phosphorylated AKT; (B–C) Quantitative analysis (n = 3). Results are expressed as mean ± SD. Significant difference (one-way ANOVA): *P < 0.05, **P < 0.01, and ***P < 0.001.


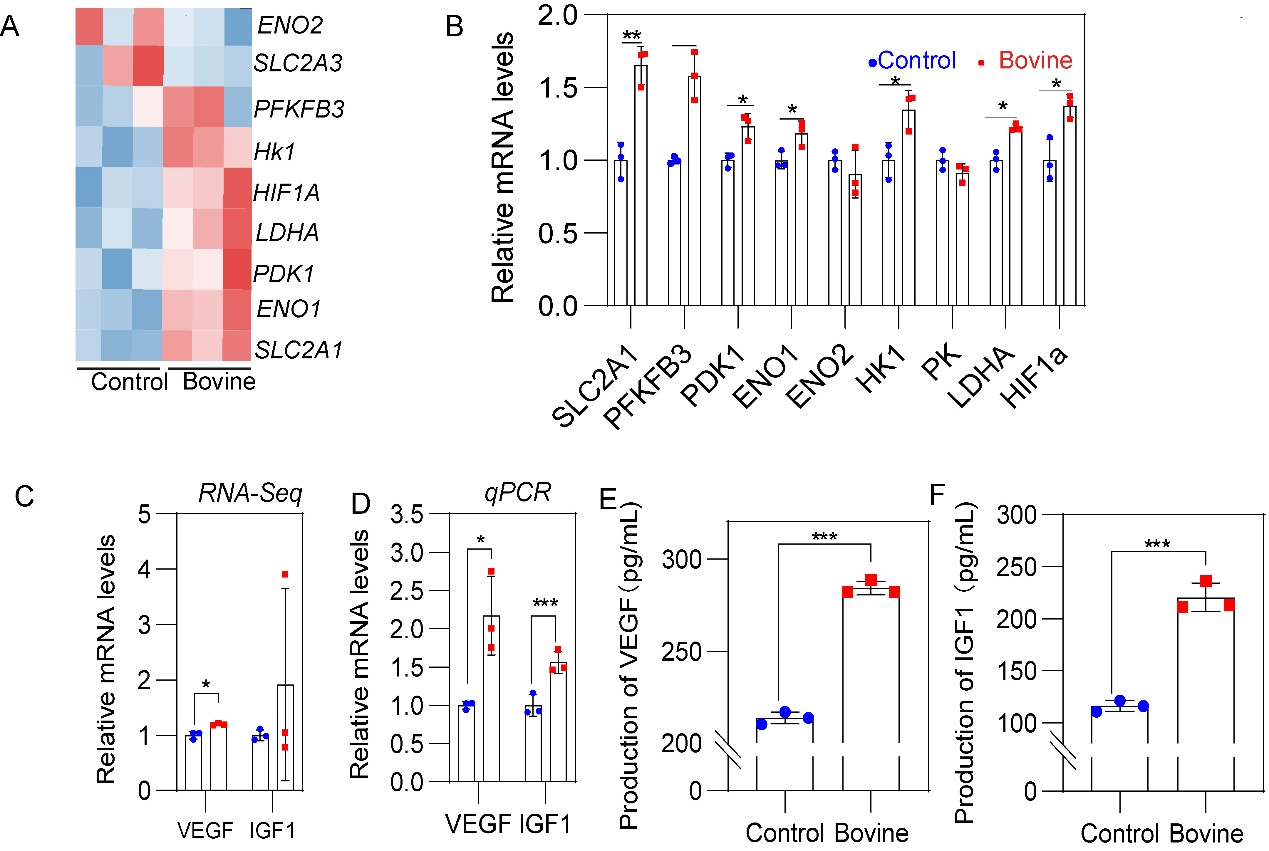


Figure S17. **A)** Heatmap related to glycolysis of gene expression levels after bovine type I collagen treatment. **B)** mRNA levels of glycolysis-related genes after bovine type I collagen treatment (n = 3). **C-D)** Expression of *VEGFA* and *IGF1* in MSCs after bovine type I collagen treatment by RNA-seq and qPCR. **E-F)** ELISA measurements of *VEGFA* and *IGF1* levels (n = 3). Results are expressed as mean ± SD. Significant difference (one-way ANOVA): *P < 0.05, **P < 0.01, and ***P < 0.001.


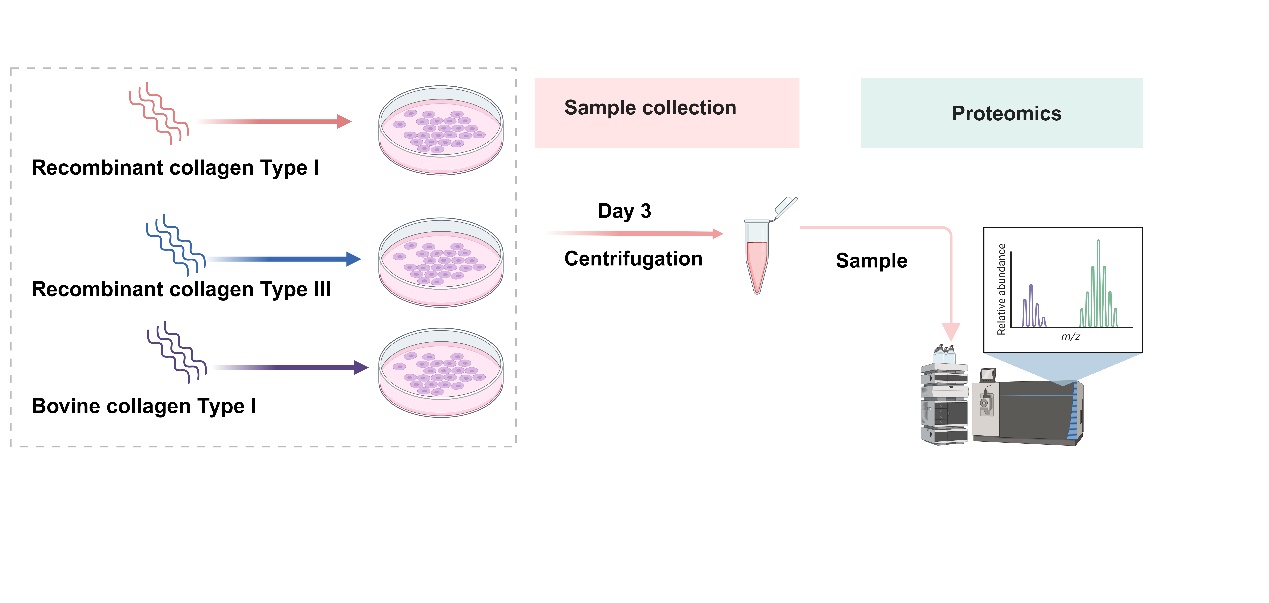


Figure S18. schematic of proteomic profiling of secreted proteins from MSCs treated with collagen for 3 days (n = 3)


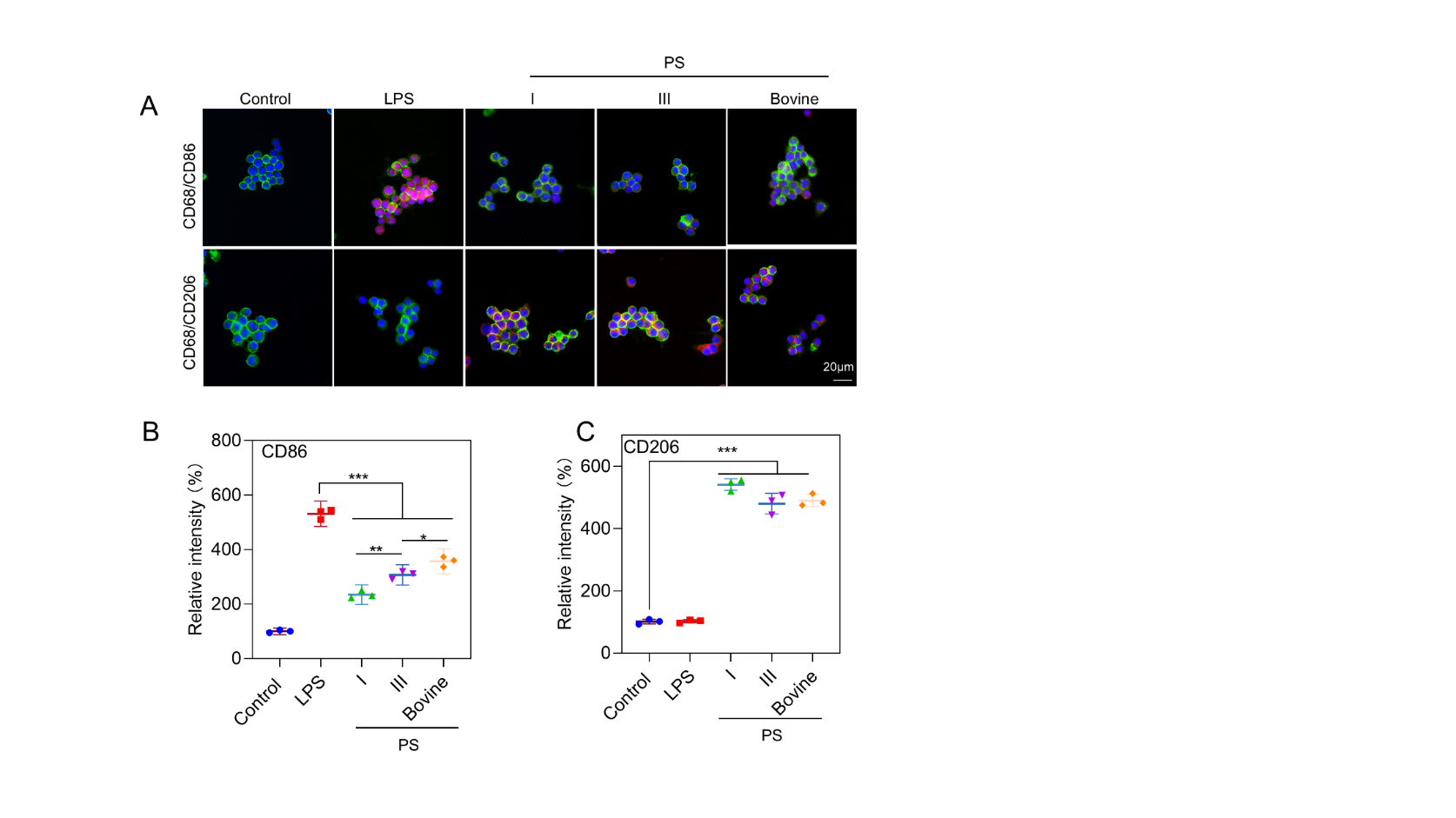


Figure S19. The interaction between MSCs and collagen in the co-culture system promoted M1-M2 macrophage polarization. A) Immunofluorescence staining of macrophage CD68, CD86, and CD206. B) Quantitative analysis of CD86 (n=3). C) Quantitative analysis of CD206 (n=3). Results are expressed as mean ± SD. Significant difference (one-way ANOVA): *P < 0.05, **P < 0.01, and ***P < 0.001.


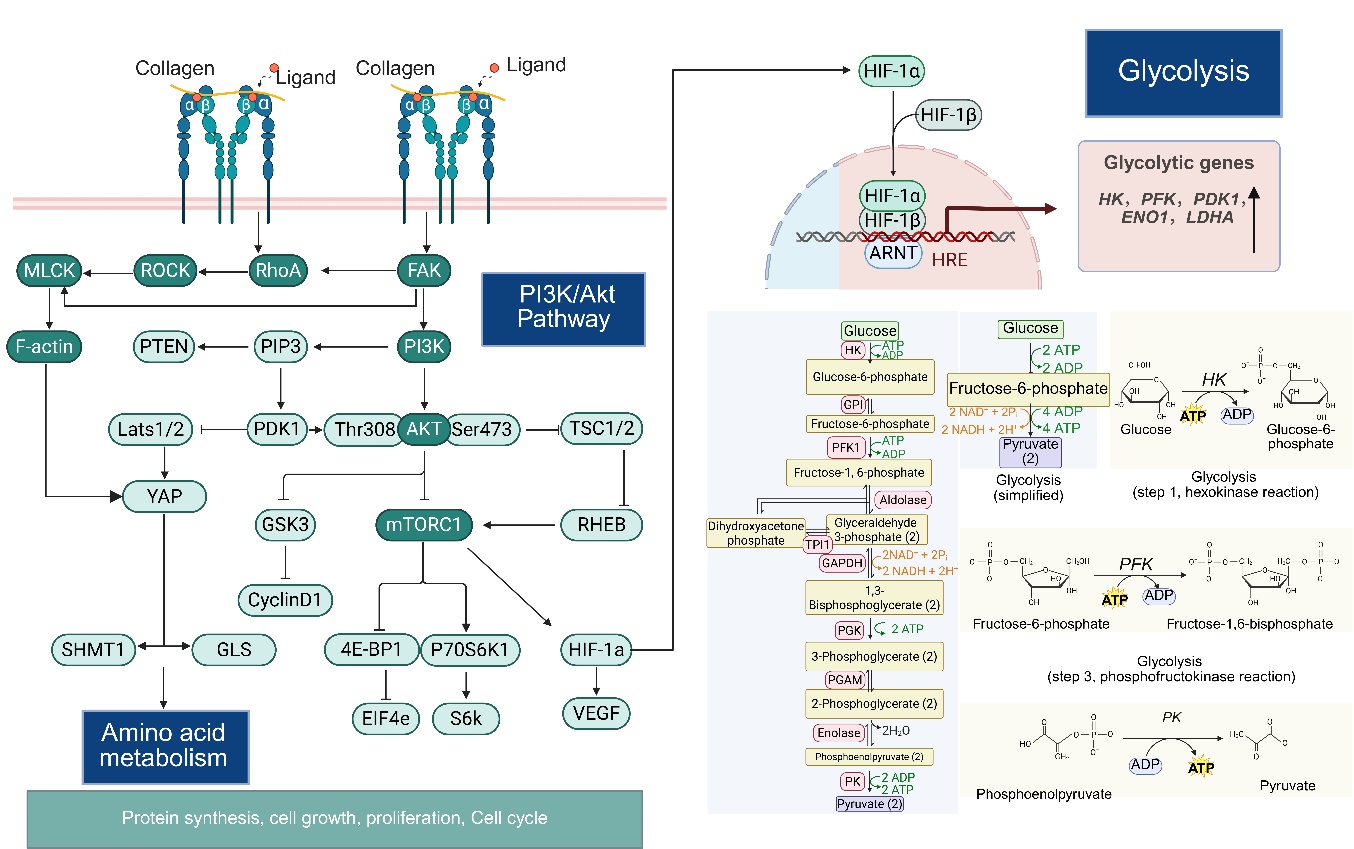


Figure S20. Functional interaction network illustrating the connections between PI3K/AKT, HIF-1α, glycolysis, and other enriched pathways.


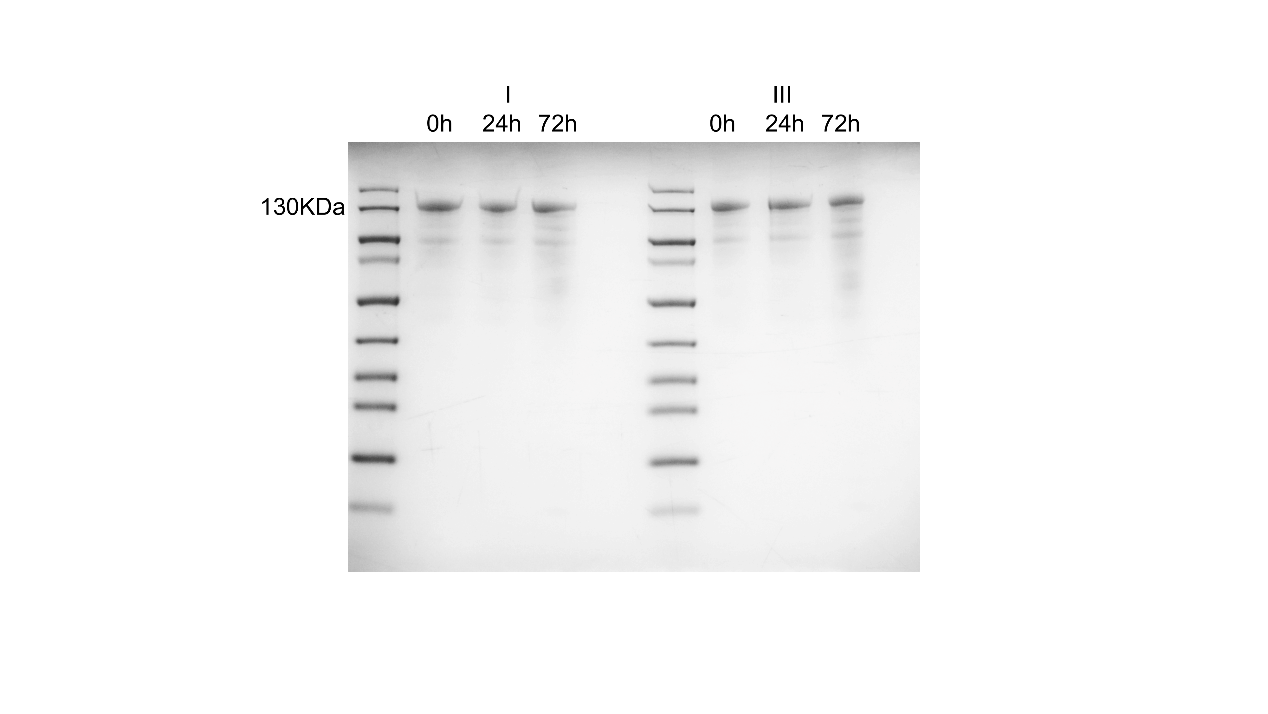


Figure S21. SDS-page images of recombinant collagen under cell culture conditions for 24h and 72h.

| Table S1. List of primers for qPCR analysis. | | |
| --- | --- | --- |
| Gene | Forward & Reverse | Sequence (5’- 3’) |
| PHGDH | Forward | CTTACCAGTGCCTTCTCTCCAC |
|  | Reverse | GCTTAGGCAGTTCCCAGCATTC |
| GOT2 | Forward | CCAAGGCTTTGCCAGTGGTGAT |
|  | Reverse | AGTGAAGGCTCCTACACGCTCA |
| GPT2 | Forward | ATCCTCACGCTGGAGTCCATGA |
|  | Reverse | ATGTTGGCTCGGATGACCTCTG |
| PYCR1 | Forward | TGCCTTGCATGTGCTGGAGAGT |
|  | Reverse | GCTTCACCTTGTCCAGGATGGT |
| GLUD1 | Forward | CTCCAGACATGAGCACAGGTGA |
|  | Reverse | CCAGTAGCAGAGATGCGTCCAT |
| GLS | Forward | CAGAAGGCACAGACATGGTTGG |
|  | Reverse | GGCAGAAACCACCATTAGCCAG |
| CBS | Forward | CATTGCCAGGAAGCTGAAGGAG |
|  | Reverse | CATTGCCAGGAAGCTGAAGGAG |
| MTR | Forward | CCAACTTGTCCTTCTCCTTCCG |
|  | Reverse | CATACACAGGGAGGTTTCCAGC |
| CTH | Forward | CTCACTGTCCACCACGTTCAAG |
|  | Reverse | CAGTGGCTGCTAAACCTGAAGC |
| SHMT1 | Forward | TGAACACTGCCATGTGGTGACC |
|  | Reverse | CTCTTTGCCAGTCTTGGGATCC |
| SHMT2 | Forward | GCCTCATTGACTACAACCAGCTG |
|  | Reverse | ATGTCTGCCAGCAGGTGTGCTT |
| GLDC | Forward | GCTTGGTGAGAATGATGCCTGG |
|  | Reverse | CAGATGTTGCTGGTAGCCTTGTC |
| MAT2A | Forward | CTGGCAGAACTACGCCGTAATG |
|  | Reverse | GTGTGGACTCTGATGGGAAGCA |
| AHCY | Forward | ATCCTCAAGGTGCCTGCCATCA |
|  | Reverse | CGGCAATCATCACATCTGTGGC |
| HIF1A | Forward | TTCCAGTTACGTTCCTTCGATCA |
|  | Reverse | TTTGAGGACTTGCGCTTTCA |
| ITGA1 | Forward | CTCCTCACTGTTGTTCTACGCT |
|  | Reverse | ATCCAAACATGTCTTCCACCG |
| ITGA2 | Forward | CCTACAATGTTGGTCTCCCAGA |
|  | Reverse | AGTAACCAGTTGCCTTTTGGATT |
| ITGA3 | Forward | CCCACCTGGTGTGACTTCTT |
|  | Reverse | TCCCTGGAGGTGGGTAGC |
| ITGA5 | Forward | TGCCGAGTTCACCAAGACTG |
|  | Reverse | TGCAATCTGCTCCTGAGTGG |
| ITGA6 | Forward | CAACTTGGACACTCGGGAGG |
|  | Reverse | ACGAGCAACAGCCGCTT |
| ITGB1 | Forward | CCGCGCGGAAAAGATGAATTT |
|  | Reverse | AGCAAACACACAGCAAACTGA |
| ITGB3 | Forward | TTGGAGACACGGTGAGCTTC |
|  | Reverse | GCCCACGGGCTTTATGGTAA |
| PI3K | Forward | ACTGCCGAGAGATTTTCCCAC |
|  | Reverse | TCACTCATCTGTCGCAGGCA |
| AKT1 | Forward | GCTCAGCCCACCCTTCAAG |
|  | Reverse | GCTGTCATCTTGGTCAGGTGGT |
| TSC1 | Forward | CTGGACAGACTGATACAGCAGG |
|  | Reverse | TGCGGATCTCATCTGAAGGAGG |
| Rheb | Forward | CTATCTTTCCTCAGACATACTCCA |
|  | Reverse | CACCATATCCAACAATTTGCCATG |
| 4EBP1 | Forward | CACCAGCCCTTCCAGTGATGAG |
|  | Reverse | CCTTGGTAGTGCTCCACACGAT |
| raptor | Forward | CTGACCTATTCACCTCCTGCCT |
|  | Reverse | GACCTCATAATCCTTTCCGCC |
| S6K1 | Forward | TATTGGCAGCCCACGAACACCT |
|  | Reverse | GTCACATCCATCTGCTCTATGCC |
| LKB1 | Forward | CTACTGAGGAGGTTACGGCACA |
|  | Reverse | ACGCTGTCCAGCATTTCCTGCA |
| PFK2 | Forward | GGCAGGAGAATGTGCTGGTCAT |
|  | Reverse | CATAAGCGACAGGCGTCAGTTTC |
| MLX | Forward | ACACTCAGGCTGAGCAGAAGAG |
|  | Reverse | GAGCCAATGGAGAAGTCCTGCT |
| ACC1 | Forward | ATCGCCCTGTGGATGACTGA |
|  | Reverse | GAGACAGCCAGGAGAAATCAAAC |
| TXNIP | Forward | CAGCAGTGCAAACAGACTTCGG |
|  | Reverse | CTGAGGAAGCTCAAAGCCGAAC |
| PGK | Forward | CCGCTTTCATGTGGAGGAAGAAG |
|  | Reverse | CTCTGTGAGCAGTGCCAAAAGC |
| PK | Forward | GCATCTACCTGGACACGGACTT |
|  | Reverse | ATGCACAGCGCCATGAACTCGT |
